# From Algorithms to Clinics: Recent Progress in AI for Scoliosis Diagnosis and Management

**DOI:** 10.3390/jcm15166361

**Published:** 2026-08-18

**Authors:** Róża Kosińska, Artur Fabijan, Robert Fabijan, Laura Kosińska, Emilia Nowosławska, Krzysztof Zakrzewski, Bartosz Polis

**Affiliations:** 1 Department of Neurosurgery, Polish-Mother’s Memorial Hospital Research Institute, 93-338 Lodz, Poland; artur8944@wp.pl (A.F.); emilia.nowoslawska@iczmp.edu.pl (E.N.); krzysztof.zakrzewski@iczmp.edu.pl (K.Z.); jezza@post.pl (B.P.); 2 Independent Researcher, Luton LU2 0GS, UK; robert.f.fabijan@gmail.com; 3 Institute of Turbomachinery, Lodz University of Technology, 90-924 Lodz, Poland; laura.w.kosinska@gmail.com

**Keywords:** scoliosis, adolescent idiopathic scoliosis, artificial intelligence, machine learning, deep learning, Cobb angle, spinal deformity, predictive models, large language models, surgical planning

## Abstract

Scoliosis is a complex three-dimensional spinal deformity that requires early detection, accurate radiological assessment, individualized treatment planning, and long-term monitoring. In recent years, artificial intelligence (AI) has emerged as a promising tool across multiple stages of scoliosis management, including screening, automated imaging analysis, deformity classification, prediction of progression, surgical planning, postoperative outcome assessment, and patient education. This narrative review summarizes current and emerging applications of AI in scoliosis, with particular emphasis on studies published after 2023. Deep learning algorithms, including convolutional neural networks, U-Net-based architectures, transformer models, and generative approaches, have demonstrated high accuracy in automated Cobb angle measurement, vertebral segmentation, coronal and sagittal parameter assessment, and radiation-free screening using surface topography or smartphone-based photographs. Machine learning models have also shown potential in predicting curve progression, treatment response, risk of postoperative complications, and patient-reported outcomes by integrating radiological, clinical, biomechanical, and, increasingly, multimodal data. In parallel, large language models and generative AI tools are being investigated for patient education, communication support, readability improvement, and research hypothesis generation. Despite these advances, important limitations remain, including limited external validation, dataset heterogeneity, potential algorithmic bias, insufficient interpretability, and incomplete integration into clinical workflows. Moreover, while AI systems show strong performance in automated measurement and screening tasks, their role in complex therapeutic decision-making, such as Lenke classification, fusion-level selection, and autonomous surgical planning, remains experimental. Overall, AI has the potential to improve the precision, efficiency, and personalization of scoliosis care; however, prospective multicentre studies, transparent reporting, explainable model design, and regulatory validation are essential before widespread clinical implementation.

## 1. Introduction

**Figure 1:**
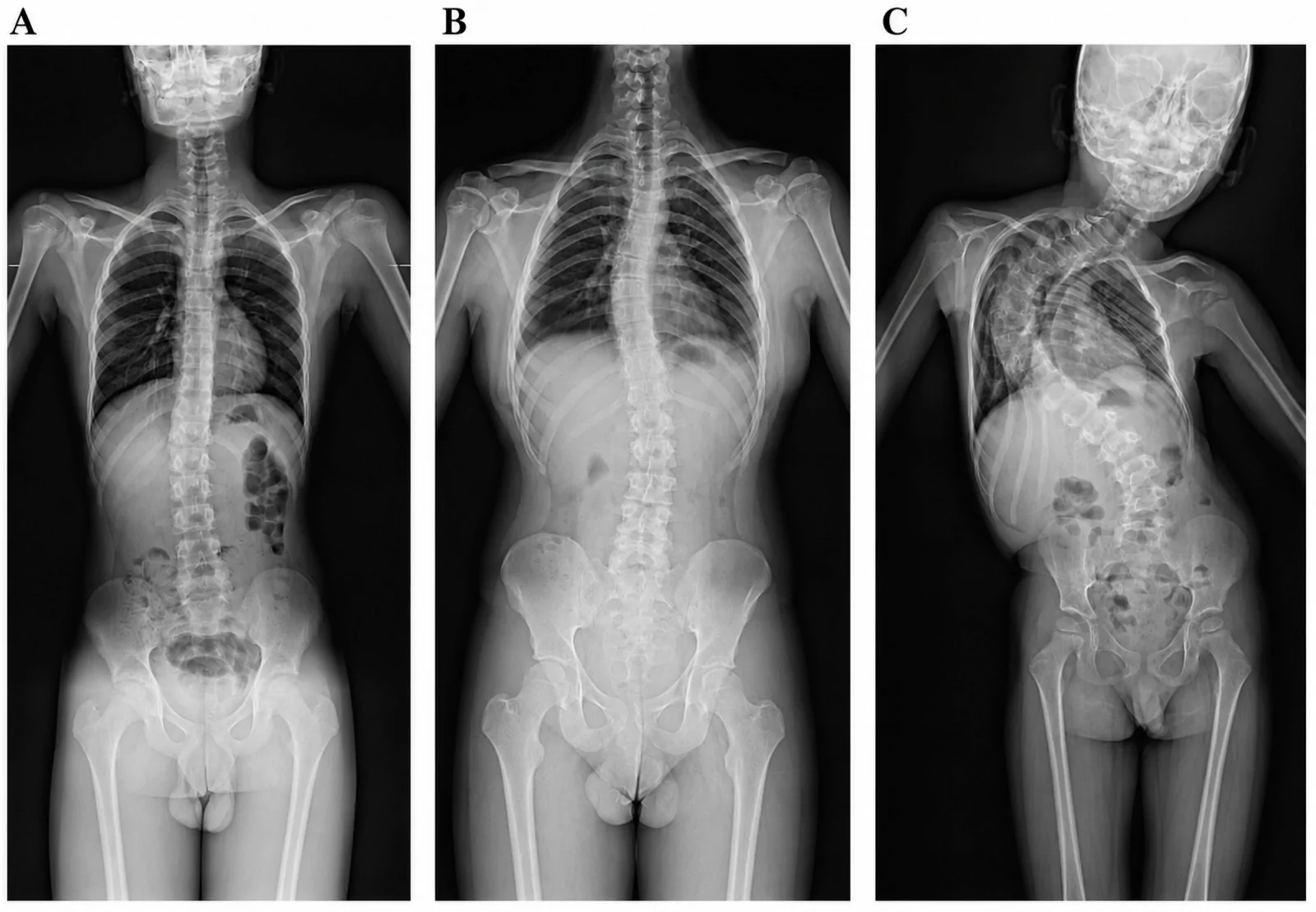
Representative radiographs illustrating different degrees of scoliosis severity. (**A**) Mild scoliosis with a Cobb angle of 13°. (**B**) Moderate scoliosis with a Cobb angle of 26°. (**C**) Severe scoliosis with a Cobb angle of 86°. The panels demonstrate progressive spinal deformity severity based on increasing coronal curvature magnitude.

Scoliosis is a three-dimensional deformity of the spine, traditionally defined by a lateral curvature greater than 10° measured using the Cobb angle [1]. Adolescent idiopathic scoliosis (AIS) is the most common form in children and adolescents, affecting approximately 2–4% of the adolescent population and occurring more frequently in girls than in boys [2]. Although many curves remain mild, progressive deformity may lead to pain, functional impairment, cosmetic concerns, reduced quality of life, and, in severe cases, cardiopulmonary compromise [1,3]. Accurate diagnosis, reliable monitoring, and timely treatment decisions are therefore central to effective scoliosis management (Figure 1).

Current clinical practice relies heavily on radiographic assessment, specialist interpretation, and longitudinal follow-up [4,5]. These processes are clinically effective but have important limitations. The Cobb angle measurement is subject to interobserver variability, repeated radiographs expose children to cumulative ionizing radiation, and complex treatment decisions require integration of multiple anatomical, developmental, functional, and patient-specific factors [1,6,7,8]. These challenges have created growing interest in artificial intelligence (AI) as a tool to improve measurement reproducibility, reduce workload, support early screening, and assist individualized decision-making [9].

Recent AI research in scoliosis has developed along several complementary directions. In imaging, deep learning models have been used to automate vertebral segmentation, landmark detection, Cobb angle measurement, sagittal alignment assessment, and three-dimensional deformity analysis. Radiation-free approaches, including surface topography, smartphone-based back photographs, ultrasound, and other non-invasive modalities, are being investigated as tools for screening and follow-up. In parallel, predictive models have been designed to estimate curve progression, brace response, postoperative complications, surgical correction, and patient-reported outcomes. More recently, large language models have also been explored as tools for patient education, communication support, readability improvement, and clinical information synthesis [10,11,12].

However, the translation of AI into routine scoliosis care remains limited. Many published models are retrospective, single-centre, and trained on relatively homogeneous datasets. External validation is often incomplete, reporting standards vary, and model interpretability remains a major barrier to clinical trust. In addition, AI systems designed for one task, such as Cobb angle measurement, may not generalize to broader clinical decision-making, where the relevant question is not only whether the model is accurate, but whether it improves patient outcomes, workflow efficiency, safety, and shared decision-making.

A comprehensive review published in 2023 summarized earlier applications of AI in scoliosis, including screening, diagnosis, treatment planning, intraoperative support, and outcome prediction [13]. Since then, the field has expanded rapidly, particularly in automated radiographic measurement, radiation-free screening, multimodal prediction, and generative AI. An updated synthesis is therefore needed to clarify where AI is clinically mature, where evidence remains preliminary, and which methodological gaps must be addressed before implementation.

This review aims to summarize recent progress in AI for scoliosis diagnosis and management, with emphasis on literature published after 2023. The review focuses on five main areas: automated imaging assessment, screening and early detection, predictive modelling, surgical and postoperative applications, and language-model-based tools for education and communication. Particular attention is given to clinical relevance, methodological limitations, and the requirements for safe translation into scoliosis care.

## 2. Methods

The PubMed database was initially searched on 23 June 2026 and the search was updated on 31 July 2026 to identify studies published from 1 January 2023 to 31 July 2026 concerning applications of artificial intelligence in the screening, diagnosis, imaging assessment, monitoring, prognosis, treatment planning, surgical management, postoperative care, patient education, and research of scoliosis. The search strategy combined the term “scoliosis” with “artificial intelligence,” “machine learning,” “deep learning,” “neural network,” “computer vision,” “large language model,” “generative artificial intelligence,” and “natural language processing.” Searches were conducted within titles and abstracts. Additional relevant studies, including earlier foundational publications, were identified through manual screening of the reference lists of eligible articles and related reviews.

Studies were considered eligible if they evaluated an artificial intelligence- or machine-learning-based method applied to scoliosis screening, diagnosis, automated imaging assessment, deformity classification, prediction of disease progression or treatment outcomes, surgical planning, postoperative assessment, or patient education and communication. Publications were excluded if they did not specifically concern scoliosis, did not evaluate an AI- or machine-learning-based application, duplicated previously identified reports, or provided insufficient information to determine the clinical task, methodological approach, or principal findings.

Titles and abstracts were initially screened for relevance. Full-text publications were subsequently reviewed whenever they were available, whereas studies accessible only as abstracts were assessed on the basis of the information explicitly reported in the abstract. To identify additional relevant publications that might not have been retrieved through the primary search, the reference lists of the selected articles were also examined. The included studies were then organized into five thematic categories according to their predominant clinical application: imaging and automated measurement, screening and classification, prediction and prognosis, surgical and postoperative applications, and large language models and patient-oriented tools.

To strengthen the methodological critique during manuscript revision, we performed a structured qualitative appraisal of the included primary studies. The assessment was adapted to the design and intended purpose of each study rather than applying a single appraisal framework uniformly to all AI applications. Studies developing or evaluating diagnostic and prognostic models were examined with reference to relevant domains from TRIPOD + AI and PROBAST + AI, including participant and data-source selection, unit of analysis, reference-standard quality, data partitioning, overfitting, calibration, discrimination, external and temporal validation, and clinical applicability.

Where applicable, prospective, interventional, and implementation studies were additionally interpreted in relation to CONSORT-AI, SPIRIT-AI, DECIDE-AI, and the FUTURE-AI framework. The appraisal was based on full-text publications whenever available. When only an abstract was accessible, the assessment was restricted to information explicitly reported in the abstract. Because of the heterogeneous study designs and incomplete methodological reporting, formal study-level PROBAST + AI ratings were not assigned. Instead, methodological strengths, limitations, and reporting uncertainties were summarized descriptively in the tables and considered when assessing clinical readiness.

## 3. AI in Imaging and Automated Measurement of Spinal Deformity

Artificial intelligence has been most extensively investigated in scoliosis imaging, where it has been applied to automated curve detection, vertebral segmentation, landmark localisation, Cobb angle measurement, sagittal balance assessment, and three-dimensional deformity analysis [1,13]. This area currently represents one of the most clinically mature applications of AI in scoliosis, because many of these tasks are repetitive, time-consuming, and affected by interobserver variability. Deep learning architectures, including convolutional neural networks (CNNs), U-Net-based segmentation models, ResNet-derived networks, pose-estimation algorithms, transformer-based models, and generative adversarial networks (GANs), have been used across different imaging modalities [14,15,16]. Deep learning methods have been applied to scoliosis assessment across several imaging modalities, including computed tomography [15], magnetic resonance imaging [14,16], and photographic assessment of the back [17].

AI-based measurement systems may support specialist assessment by improving the speed, consistency, and reproducibility of radiographic spinal deformity evaluation [1,18]. In particular, automated measurement may reduce variability in the Cobb angle assessment, support large-scale screening, facilitate longitudinal monitoring, and provide additional quantitative parameters that are difficult to obtain manually in routine practice. The methodological characteristics, validation strategies, principal limitations, and clinical readiness of the included systems are summarised in Supplementary Table S1.

### 3.1. Radiation-Free Screening Methods

One important direction in AI-based scoliosis imaging is the development of radiation-free methods for early detection and follow-up. This is particularly relevant in children and adolescents, who may require repeated assessment during growth. Surface topography, back photography, smartphone-based image analysis, and ultrasonography have therefore been investigated as alternatives or complements to radiographic monitoring.

Three-dimensional markerless surface topography of the back, analysed using CNNs, has been reported to distinguish individuals with scoliosis from healthy controls with high diagnostic performance. One study reported an accuracy of approximately 95%, with 97% sensitivity and 90% specificity, for detecting scoliosis based on trunk asymmetry [19]. This suggests that AI-assisted surface topography may be useful as a screening tool, particularly because it avoids ionising radiation. However, such models often function as binary classifiers and may not provide reliable information on curve magnitude or severity. Their performance also requires further validation in broader clinical and community-based populations.

Two-dimensional photographs of the back have also been explored for AI-assisted scoliosis assessment. A Dual Attention U-Net model was used to segment the back contour and calculate an asymmetry index for scoliosis detection and severity estimation. The model achieved an AUC of 0.93 for scoliosis detection and reported sensitivity, specificity, and precision above 90% for identifying severe scoliosis [10]. These findings suggest that photographic assessment may be useful for inexpensive and scalable screening. However, image quality, patient positioning, obesity, mild deformity, and uncontrolled acquisition conditions may reduce performance, especially outside research settings.

Smartphone-based systems may further improve accessibility. The ScolioNets deep learning model was incorporated into the AlignProCARE mobile application to classify scoliosis severity and curve type from smartphone photographs of the back, using radiographic assessments as the reference standard. In a prospective validation cohort, the model demonstrated promising sensitivity and negative predictive values for identifying patients requiring follow-up or consideration for surgery, indicating potential utility as an accessible triage and monitoring tool [20]. This could help identify patients who need radiographic assessment while reducing unnecessary imaging in low-risk cases. However, this technology should still be interpreted cautiously because validation has been limited by factors such as device type, image acquisition conditions, and the range of patient body habitus included in testing.

Ultrasonography is a radiation-free imaging modality that may be useful for scoliosis assessment and follow-up. However, interpretation of spinal ultrasound images can be challenging because of acoustic shadows, speckle noise, indistinct boundaries of bony structures, and variability in image acquisition. AI-based segmentation may help address these limitations and improve visualisation of spinal deformity. For example, the UGBNet architecture, which incorporates spatial and channel attention mechanisms together with multiscale feature fusion, achieved a Dice coefficient of 74.2% for the segmentation of bony structures on spinal ultrasound images [21]. This remains less accurate than radiographic assessment, but it represents a step towards non-ionising follow-up tools, particularly for children undergoing conservative treatment.

### 3.2. Automated Cobb Angle Measurement on Radiographs

Automated Cobb angle measurement on conventional radiographs is currently one of the most developed applications of AI in scoliosis imaging. The Cobb angle remains the standard parameter for diagnosis, severity grading, treatment selection, and follow-up. However, manual measurement is time-consuming and subject to interobserver variability, commonly estimated at approximately 3–5°. AI-based methods have therefore been developed to identify vertebral structures, select end vertebrae, and calculate Cobb angles automatically.

In one study, a model combining a ResNet34 backbone with elements of the U-Net architecture achieved a mean absolute difference of 2.15° for coronal Cobb angle measurements on anteroposterior radiographs and demonstrated excellent agreement with specialist-derived reference measurements, with an ICC of 0.985. The same model also showed promising performance in the assessment of sagittal parameters, including proximal thoracic kyphosis, mid-thoracic kyphosis, and thoracolumbar sagittal alignment, with an overall mean absolute difference of 2.72° and an ICC of 0.927 [22]. These results suggest that combined feature-extraction and segmentation architectures may support reproducible assessment in both coronal and sagittal planes. However, such findings should be interpreted in relation to the dataset, imaging protocol, and reference-standard methodology used in the original study.

Further work has shown that Cobb angle measurement can be fully automated without manual selection of the spinal region or end vertebrae. Chen et al. evaluated a commercially available AI system using 196 anteroposterior or posteroanterior whole-spine radiographs. Following comparison with a reference standard established by four radiologists, 194 curves were included in the final performance analysis. The software achieved 88.6% accuracy in end-vertebra selection, demonstrated a mean difference of 0.16° and a mean absolute deviation of 2.47° from the reference measurements, and achieved an ICC of 0.97. These results indicate that fully automated Cobb angle measurement can achieve high agreement with expert assessment in selected datasets [23]. These results indicate that automated measurement can approach expert-level reproducibility in selected datasets.

More recent multicentre studies have begun to address the challenge of generalisability. The SPARC system achieved a curve-detection rate of 94.0% and a mean absolute error of 3.01°, whereas SpineHRNet+, combined with pixel-intensity transformation, maintained errors below 4° and R^2^ values above 0.90 across five independent external validation cohorts [24,25]. These studies are particularly important because they evaluate model performance across heterogeneous imaging sources and clinical settings, which is essential before broader clinical implementation.

Agreement metrics require clinical interpretation. A high intraclass correlation coefficient can be driven by a wide range of curve magnitudes and does not exclude systematic bias, incorrect end-vertebra selection, or clinically important errors in individual patients. Mean absolute error should therefore be interpreted together with limits of agreement, failure rates, and the proportion of measurements that cross clinically relevant severity or treatment thresholds. An average error of 2–3° may be acceptable for many follow-up examinations, but the same error can change classification or management in a patient whose Cobb angle lies close to a decision boundary. Accordingly, automated measurement systems should report Bland–Altman analyses, subgroup performance, uncertainty estimates, and the frequency with which clinician correction is required, rather than relying on ICC or mean error alone.

Different technical strategies have been used for automated radiographic assessment. Some models rely on segmentation of vertebral bodies or spinal regions, whereas others identify anatomical landmarks, such as vertebral corners, and use their geometric relationships to calculate Cobb angles. Maeda et al. developed a two-stage landmark-detection approach in which a ResNet34-based network first identified the thoracolumbar region of interest and a second network localised the four corners of each vertebral body from T1 to L5. The model was evaluated on standing, supine, side-bending, and brace radiographs and demonstrated good-to-excellent agreement with clinician measurements in most conditions, with an ICC of 0.973 for the major curve on standing radiographs. The AI-derived Cobb angles were, on average, approximately 1.7–2.2° lower than clinician measurements, and the magnitude of this difference did not increase with curve severity [26].

Landmark detection has also been applied to sagittal assessment. Kang et al. developed an RTMpose-based model that localised 38 anatomical keypoints on lateral full-spine radiographs and used them to automatically calculate 15 sagittal alignment parameters. Compared with the reference standard, the proportion of correctly predicted keypoints ranged from 78% to 100% within a 3-mm threshold and from 91% to 100% within a 4-mm threshold. The resulting measurements demonstrated high agreement with specialist-derived reference values, with ICCs ranging from 0.892 to 0.991. Mean absolute errors ranged from 0.22° to 3.32° for angular parameters, including thoracic kyphosis and lumbar lordosis, while the mean absolute error for the sagittal vertical axis was 1.16 mm. These findings indicate that landmark-based models may extend automated spinal assessment beyond coronal Cobb angle measurement to a comprehensive evaluation of sagittal alignment [27].

Modified segmentation networks have also been used to measure multiple scoliosis-related radiographic parameters simultaneously. Liu et al. applied a multi-resolution VB-Net–based system to full-length anteroposterior radiographs from 2092 patients with adolescent idiopathic scoliosis. The system automatically measured 10 coronal-plane parameters, including the Cobb angle, coronal balance distance, clavicle angle, radiographic shoulder height, T1 tilt, coronal pelvic tilt, leg-length discrepancy, sacral slope, apical vertebral translation, and thoracic trunk shift [28]. Such multiparametric analysis may support a more comprehensive characterisation of spinal deformity than Cobb angle measurement alone. However, because the study was retrospective and single-centre and excluded patients with a history of spinal surgery, independent validation against expert measurements across external centres, imaging systems, and postoperative cases remains necessary before broader clinical implementation.

### 3.3. Biplanar and Three-Dimensional Analysis

Anteroposterior and lateral whole-spine radiographs are routinely used for the radiographic diagnosis and assessment of scoliosis. Recent algorithms have extended automated analysis beyond coronal Cobb angle measurement by evaluating both coronal and sagittal spinal alignment within a single workflow. Xie et al. developed a deep learning model based on a ResNet34 backbone incorporating features of the U-Net architecture for automated landmark detection and measurement on paired anteroposterior and lateral radiographs. The model identified vertebral corners from T1 to L5 and automatically calculated multiple coronal Cobb angles together with proximal thoracic kyphosis, mid-thoracic kyphosis, and thoracolumbar sagittal alignment. It achieved an accuracy of 97.2% for scoliosis diagnosis and an overall accuracy of 94.5% for severity classification. However, sensitivity for identifying severe scoliosis, defined as a Cobb angle greater than 45°, was lower at 88.2%, indicating that approximately 12% of severe cases were not correctly classified as severe. This limitation is clinically relevant because misclassification near treatment thresholds could influence referral or treatment planning and supports the use of the model as an assistive rather than autonomous tool. The authors attributed the reduced performance in severe deformities to marked vertebral rotation and tilt and to poorly defined vertebral endplate morphology, which complicated vertebral-corner localisation. Moreover, the study was retrospective and single-centre, used an internal random train–test split, and excluded poor-quality images, postoperative radiographs, severe pelvic tilt, and examinations performed in braces; therefore, its generalisability to broader clinical populations remains uncertain [22].

One potential approach to improving automated Cobb angle measurement is to incorporate structural information about adjacent vertebrae. Du et al. developed the Landmark and Adjacent Offset Detection Network (LAD-Net), which predicted vertebral centre and corner landmarks together with offset vectors describing their spatial relationships. The proposed Adjacent Centre Iterative Correction module used information from neighbouring vertebral centres to correct incorrectly localised landmarks, while the Corner Feature Optimization and Fusion module refined vertebral-corner detection. The resulting landmarks were used to calculate Cobb angles and support automated Lenke classification [29]. More broadly, recent AI systems can extract multiple coronal and sagittal alignment parameters from spinal radiographs, extending automated assessment beyond Cobb angle measurement alone [27,28].

Lang et al. developed a fully automated deep learning pipeline based on modified U-Net architectures with inception modules to calculate coronal and sagittal spinopelvic parameters from paired anteroposterior and lateral EOS radiographs. The system automatically measured Cobb angles, thoracic kyphosis, lumbar lordosis, sagittal vertical axis, sacral slope, pelvic tilt, and pelvic incidence. However, complete segmentation and prediction of all parameters were achieved in only 45% of the previously unseen test cases, limiting the pipeline’s readiness for routine clinical use. Among successfully processed cases, the mean absolute deviation for Cobb angle measurement was 3.3°, and 80% of predictions were within ±5° of the reference values. Most sagittal angular measurements were also within ±5° of the reference standard, while 85% of SVA measurements were within an acceptable margin of ±5.7 mm. Performance varied between parameters, with good-to-excellent agreement for Cobb angles, lumbar lordosis, SVA, and pelvic tilt, but only moderate agreement for thoracic kyphosis and sacral slope. The principal limitation was incomplete segmentation, particularly in the thoracic region, where overlapping ribs, scapulae, and humeri complicated vertebral identification. Further improvements in segmentation robustness are therefore required, especially in patients with severe pathology, high BMI, or spinal instrumentation [30].

In the context of three-dimensional spinal assessment, the application of AI to computed tomography is of particular interest because volumetric imaging enables a more comprehensive evaluation of spinal curvature and vertebral alignment than a single two-dimensional projection, although manual analysis of such data is labor-intensive. Li et al. developed SpineCurve-net, a deep learning framework combining a 3D U-Net for spinal segmentation with NURBS-net for three-dimensional curve fitting and automated Cobb angle estimation from preoperative CT images. In a cohort of 116 patients with adolescent idiopathic or degenerative scoliosis, the predicted 3D Cobb angle showed a strong correlation with surgeon-annotated Cobb angles measured on standing posteroanterior radiographs (r = 0.983), with a mean absolute error of 2.4° and Bland–Altman limits of agreement ranging from −4.0° to 6.2°. The authors suggested that the patient-specific three-dimensional information provided by the model could support preoperative assessment and surgical planning. However, the study was retrospective and single-centre, and the internal validation cohort included only 27 patients with adolescent idiopathic scoliosis. Moreover, CT and radiographic measurements were acquired in different patient positions, and the predicted 3D angles were not compared with an independent three-dimensional reference standard, limiting conclusions regarding generalisability and true 3D measurement accuracy. The authors also proposed that predicting a limited number of NURBS control points and knots may reduce curve overfitting, while restricting the dataset to scoliosis cases increased the specificity of model training for spinal deformity [15].

Opportunities for AI application are also emerging in magnetic resonance imaging. Routine sagittal lumbar MRI is not conventionally used for coronal Cobb angle measurement because it does not provide a single standing coronal projection of the entire spine and generally has a more limited field of view than whole-spine radiography. Van der Graaf et al. developed a 3D AI-based method that automatically segmented lumbar vertebrae and intervertebral discs on sagittal MRI. Three-dimensional planes fitted to the upper and lower portions of the intervertebral discs were used as approximations of the adjacent vertebral endplates, allowing all possible coronal angles to be calculated and the largest angle to be selected as the Cobb angle. In a validation cohort of 50 patients with degenerative scoliosis, the maximum angle identified by the algorithm showed good agreement with the average manual measurement of three experienced readers, with an ICC of 0.83. All three readers selected the same upper and lower vertebral levels in only 18% of cases, whereas the automated method consistently selected the largest measurable angle. When the algorithm calculated angles at the same vertebral levels selected by the readers, agreement was excellent, with ICCs ranging from 0.92 to 0.97. These findings suggest that automated MRI-based measurement may improve the consistency of Cobb angle assessment and potentially reduce the need for additional radiographs when suitable lumbar MRI scans are already available. However, the method was evaluated retrospectively in a small cohort, and MRI was acquired in the supine position and was not directly compared with standing whole-spine radiographs; therefore, its suitability for longitudinal monitoring and its interchangeability with conventional radiographic measurements remain uncertain [14].

Ultrasonography is a radiation-free and repeatable imaging modality with potential applications in scoliosis assessment. However, spinal ultrasound images are challenging to analyse because of speckle noise, acoustic shadows, low signal-to-noise ratios, indistinct boundaries, and variability in image acquisition. Jiang et al. developed UGBNet, a segmentation architecture based on a ResNet-101 backbone that incorporates dense atrous spatial pyramid pooling, multiscale feature fusion, and a global guidance block integrating spatial and channel attention. The model automatically segmented bony features, including the spinous and transverse processes, on two-dimensional spinal ultrasound images and achieved a Dice score of 74.2%, outperforming conventional U-Net and other comparison models. The resulting segmentations could subsequently be used for three-dimensional visualisation of spinal morphology. Although these findings support the potential of AI-assisted ultrasound as a radiation-free tool for scoliosis assessment, the relatively small dataset and sensitivity to poor-quality images indicate that validation in larger and more diverse clinical populations is required before its use in routine monitoring [21].

### 3.4. Generative Networks and Synthetic Images

An emerging application of artificial intelligence in scoliosis imaging is the generation of synthetic radiographs with the aim of reducing the need for additional image acquisition and the associated radiation exposure. Bassani et al. investigated a GAN-based framework for generating synthetic sagittal radiographs from coronal images in patients with adolescent idiopathic scoliosis. The model was developed using 3935 paired coronal and sagittal radiographic examinations acquired simultaneously with the EOS system. Two sequential pix2pix-based GANs were used: the first generated a sagittal projection from the coronal image, while the second enhanced the resolution and contrast of the synthetic output.

Although the generated images were visually consistent with the reference radiographs, their accuracy was generally insufficient for clinical measurement. Of 100 randomly selected test cases, only 69 synthetic images were considered assessable by both radiologists. Correlations between measurements obtained from real and synthetic images were weak to moderate for lumbar lordosis, sacral slope, and pelvic incidence, with r values of 0.52, 0.17, and 0.18, respectively. Sagittal vertical axis showed the most promising performance, with a correlation of 0.74 and a mean absolute error of 1.1 ± 0.8 cm. These findings suggest that the model reproduced global sagittal alignment more successfully than detailed angular parameters, but its performance remained insufficient for routine clinical application.

The results demonstrate the potential of generative models to reduce the need for additional sagittal radiographic acquisition; however, substantial methodological limitations remain. Patients with severe scoliosis, postoperative changes, vertebral deformities, and non-standard positioning were excluded, and nearly one-third of the evaluated synthetic images were not suitable for measurement. Further development should therefore focus on improving anatomical landmark preservation, image resolution, and robustness across more complex deformities and diverse clinical populations before synthetic radiographs can be used reliably for quantitative sagittal assessment [31].

Alternatively, semi-supervised learning has been investigated for the opportunistic detection of scoliosis on radiographs acquired for other purposes. Lee et al. developed a framework based on StyleGAN2-ADA for identifying scoliosis on posteroanterior chest radiographs. Because exact Cobb angle measurements were available for only 16% of the collected images, StyleGAN2-ADA was trained on radiographs showing scoliosis of varying severity to learn disease-related image representations. The chest radiographs were subsequently projected into the model’s latent space, and the resulting feature vectors were used to train a multilayer perceptron classifier to distinguish between normal images and those showing scoliosis.

At a threshold selected to prioritise screening sensitivity, the model achieved an AUC of 0.856, a sensitivity of 98.5%, and a negative predictive value of 95.0%. However, specificity was only 28.5%, and the positive predictive value was 57.9%, indicating that the system generated a substantial number of false-positive results. These findings suggest that the model may be useful for opportunistic screening by identifying chest radiographs that require further specialist review while rarely missing scoliosis cases. Nevertheless, the study was retrospective, the test dataset did not reflect the natural prevalence of scoliosis, and patients with other underlying abnormalities or poor-quality images were excluded. Prospective validation in representative screening populations is therefore required before clinical implementation [32].

In parallel, Xie et al. developed an automated framework combining a supervised Mask R-CNN-based module with an inference module for vertebral-body segmentation, vertebral-level identification, Cobb angle measurement, and scoliosis screening on chest radiographs. In the final test set of 491 images, the system correctly assigned the exact vertebral level in 87.6% of cases, while 99.5% of level assignments were within one adjacent vertebral segment. The mean difference between AI-derived and specialist-derived Cobb angle measurements was 0.40° ± 0.87°, with an ICC of 0.915. For binary scoliosis detection using a Cobb angle threshold of 10°, the model achieved an accuracy of 98.4%, specificity of 98.7%, sensitivity of 88.2%, and an AUC of 0.979 [33]. These findings suggest that chest radiographs acquired for other clinical indications may potentially support opportunistic scoliosis screening and automated curvature assessment. However, only 17 scoliosis cases were included in the test set, and the study used a single-source dataset without external validation. Severe scoliosis, spinal degeneration, other complex deformities, and images with spinal instrumentation were not adequately represented, limiting the generalisability of the reported performance.

Commercial deep-learning systems for automated chest radiograph analysis are also being evaluated for the simultaneous detection of multiple abnormalities. In a prospective multicentre quality-improvement study involving the qXR system, the AI module achieved an AUC of approximately 0.98 and a negative predictive value of 99.9% for identifying radiographs reported as showing scoliosis. The same system assessed a broad range of additional findings, including rib fractures, cardiomegaly, consolidation, pulmonary opacities, atelectasis, pleural effusion, and pneumothorax [34]. These findings demonstrate the potential versatility of multipathology chest radiograph algorithms and suggest that they may support the opportunistic identification of spinal curvature on images acquired for other clinical indications. However, scoliosis labels were derived from routine radiological reports rather than dedicated Cobb angle measurements or standing whole-spine radiographs; therefore, the reported performance should not be interpreted as validation of the system for definitive scoliosis diagnosis or severity assessment.

### 3.5. Other Applications of Artificial Intelligence in Spinal Deformities

Artificial intelligence applications in spinal deformity care extend beyond imaging and automated measurement. Large language models, including ChatGPT, have been evaluated as potential sources of educational information for patients and families affected by adolescent idiopathic scoliosis. Expert assessments of chatbot-generated responses have shown that these models can provide generally understandable and factually appropriate answers to common questions, but may also produce clear inaccuracies, omit clinically important details, or provide insufficiently nuanced information, particularly regarding complex topics such as surgical treatment. In a comparative evaluation, ChatGPT-4.0 received higher overall ratings than ChatGPT-3.5 and Google Bard, although no significant differences between the models were found in overall empathy scores [35]. A separate assessment of frequently asked AIS questions similarly found that ChatGPT provided generally accurate basic information but required substantial clarification or correction for some more complex management and surgical topics [36]. These findings suggest that LLMs may serve as supplementary tools for patient education, but their responses should not replace individualised counselling and should be reviewed by qualified healthcare professionals.

In addition, artificial intelligence has been investigated as a tool for improving the readability of patient education materials. In a proof-of-concept study that included educational content on scoliosis, an AI dialogue platform reduced the required reading level to approximately the sixth-grade level while retaining sufficient accuracy and clinically relevant detail following specialist review [37].

AI has also been applied to the postoperative assessment of children with early-onset scoliosis treated with magnetically controlled growing rods. Kabir et al. developed a deep learning system for automatically measuring changes in growing-rod length on posteroanterior spinal radiographs. In the final test set, the mean absolute difference between automated and manual measurements was 0.98 ± 0.88 mm, with an ICC of 0.90, indicating good agreement with manual assessment. The system generated a single rod measurement in an average of 6.1 s, suggesting potential utility for improving the efficiency and consistency of follow-up evaluation [38].

Robotic and navigation technologies are increasingly being investigated as tools for supporting pedicle screw placement in scoliosis surgery. Although these systems do not necessarily incorporate machine learning, they represent important enabling technologies that may improve the precision and reproducibility of spinal instrumentation. Akazawa et al. compared robot-assisted and navigation-assisted pedicle screw placement in patients with adolescent idiopathic scoliosis and reported a lower screw-deviation rate with robotics than with navigation alone (1.6% vs. 7.5%) [39].

In a separate study, Linden et al. compared a hybrid approach combining robotic navigation for apical screw placement with freehand insertion at the terminal levels against a freehand-only technique. After adjustment for relevant patient and surgical factors, no significant differences were observed in blood loss, curve correction, fluoroscopy time, or complication rates. However, the hybrid robotic-navigation procedure required approximately two additional minutes of operative time per screw [40]. These findings suggest that robotic navigation can provide accurate screw placement and comparable short-term surgical outcomes, although the potential benefits must be balanced against longer operative times and the additional resources required. Future systems may incorporate AI-based components for trajectory planning and intraoperative decision support, but such applications were not evaluated in these studies.

A detailed comparison of the included imaging, screening, and automated measurement studies is provided in Supplementary Table S1. The table summarises the clinical task, AI architecture, dataset characteristics, reference standard, validation strategy, principal performance metrics, methodological limitations, and current level of clinical readiness for each system. Overall, the available evidence indicates that many models achieve high agreement with expert assessment under controlled conditions, but external and temporal validation remains limited. Calibration, subgroup analysis, failure handling, and the frequency of errors crossing clinically relevant diagnostic or treatment thresholds were also infrequently reported. Furthermore, prospective workflow integration, regulatory status, and health-economic consequences were rarely evaluated. Therefore, although several systems have reached multicentre or prospective validation, most should currently be regarded as assistive tools rather than autonomous clinical solutions.

## 4. AI in Screening, Early Detection, and Classification of Scoliosis

The development of AI has substantially advanced scoliosis screening and the classification of deformity severity. Current applications range from deep learning algorithms that analyse trunk images to classical machine learning models based on clinical data. The following section reviews the most important contributions in this field, organised according to the type of input data and the intended AI application. Reported outcomes, methodological limitations, and future directions are also discussed.

### 4.1. Non-Invasive Imaging Methods: Back Surface Photographs and 3D Topography

Considerable attention has been given to radiation-free screening techniques based on body-surface imaging. Mohamed et al. developed a three-dimensional markerless surface-topography method combined with a convolutional neural network for the detection of adolescent idiopathic scoliosis. In the held-out test set, the model achieved an accuracy of 95%, a sensitivity of 97%, and a specificity of 90% in distinguishing adolescents with radiographically confirmed AIS from screen-negative participants with typically developing spines. These results were more favourable than performance values previously reported for conventional screening methods, including the Adam forward bend test and scoliometer assessment, although the methods were not compared directly within the same study population. The high sensitivity corresponded to a false-negative rate of 2.9%, suggesting a low risk of missed AIS cases under the evaluated conditions. However, the model functioned as a binary classifier and did not estimate curve magnitude or classify scoliosis severity [19].

Another radiation-free approach involves the analysis of standardised bare-back photographs. Duan et al. developed a Dual AttentionUNet model incorporating channel- and self-attention mechanisms to segment the back contour. A computer-vision-derived asymmetry index was then used to classify participants as having no scoliosis or mild, moderate, or severe deformity, with standing radiographic Cobb angle measurements serving as the reference standard. In the test set, the model achieved an AUC of 0.927 for distinguishing participants with and without scoliosis, which was comparable to the AUC of 0.921 achieved by the most experienced clinician. For severe scoliosis, defined as a Cobb angle of at least 45°, the model achieved an accuracy of 96.9%, a precision of 95.6%, a sensitivity of 92.3%, and an AUC of 0.954, closely approximating clinician performance. These findings indicate that AI-based analysis of standardised back photographs may support inexpensive and radiation-free scoliosis screening and severity stratification. However, the model did not directly predict treatment requirements, and severe-curve classification should not be equated with an indication for surgery. Moreover, the pooled random data split, controlled image-acquisition conditions, exclusion of poor-quality images, and reduced performance in patients with higher BMI or less pronounced deformities limit conclusions regarding generalisability to population-level screening [10].

Recent studies illustrate two complementary directions in AI-assisted scoliosis screening: smartphone-based three-dimensional surface topography and machine-learning models based on biomechanical features. In a prospective multicentre diagnostic study involving 236 participants, Martrenchar et al. evaluated a smartphone application that used video-based three-dimensional surface topography to estimate the major coronal curve. For scoliosis defined as a curve greater than 10°, the application achieved a sensitivity of 100% and a specificity of 89%, indicating potential utility as a preliminary triage tool for identifying children who may require confirmatory radiographic assessment [41]. However, the study was affected by potential selection bias, did not assess measurement reproducibility, and did not evaluate whether implementation of the application reduced radiographic referrals or improved clinical outcomes.

A complementary approach was investigated by Chen et al., who developed machine-learning models using foot-pressure and body-posture parameters collected from primary school students. Among the evaluated algorithms, LightGBM achieved an AUC of 0.971 and an accuracy of 92%, while SHAP analysis identified toe-out angle and centre-of-pressure sway area as the most influential predictors [42]. These findings suggest that inexpensive biomechanical measurements may complement imaging-based screening by identifying children who warrant further clinical assessment. Nevertheless, independent external and temporal validation, calibration assessment, and prospective evaluation in representative screening populations remain necessary. The organisational and health-economic consequences of both approaches, particularly the number and downstream costs of false-positive referrals, also require further investigation.

### 4.2. Radiographic Analysis: Scoliosis Detection and Automated Measurements

Other studies have focused on the automated measurement of clinically relevant coronal parameters on spinal radiographs. Berlin et al. developed a fully automated deep learning algorithm that identified the clavicles, vertebral bodies, and sacrum on anteroposterior whole-spine radiographs and calculated T1 tilt, clavicle angle, coronal balance, lumbar modifier, and proximal thoracic, thoracic, and thoracolumbar/lumbar Cobb angles. The system was retrospectively validated using 100 preoperative EOS radiographs from patients with adolescent idiopathic scoliosis, with independent measurements by two experienced physicians serving as the reference standard.

The algorithm successfully generated all parameters in all 100 cases. Agreement between AI-derived and clinician-derived measurements ranged from good to excellent, with ICCs ranging from 0.78 to 0.98. The highest agreement was observed for clavicle angle and coronal balance, whereas the lowest agreement was found for the proximal thoracic Cobb angle, with ICCs of 0.78–0.84 and comparatively large measurement errors. The authors attributed this lower performance to the short and less pronounced nature of proximal thoracic curves and to the superimposition of adjacent anatomical structures. The algorithm required approximately 15 s per image, compared with 3–7 min for manual assessment, indicating potential utility for clinical measurements and large-scale registry analyses. However, the study was retrospective and single-centre, included only preoperative AIS radiographs acquired with one EOS system, and did not validate sagittal measurements or performance on postoperative images containing spinal instrumentation [43].

AI-based landmark detection has also enabled the automated measurement of multiple sagittal alignment parameters on lateral spinal radiographs, as discussed in the preceding section [27].

### 4.3. Classification of Scoliosis Severity and Support for Therapeutic Decision-Making

Beyond detecting the presence of spinal curvature, artificial intelligence may support a more comprehensive classification of deformity phenotypes and their association with clinical outcomes. Using unsupervised clustering of coronal and sagittal radiographic parameters, Lafage et al. previously identified four distinct patterns of complex adult spinal deformity: hyper-thoracic kyphosis, moderate sagittal deformity, severe sagittal deformity, and predominantly coronal deformity.

These previously established cluster definitions were subsequently applied to a separate multicentre cohort of 1062 surgically treated patients with adult spinal deformity who completed two-year follow-up. The clusters demonstrated distinct preoperative clinical and radiographic characteristics. Patients in the hyper-thoracic kyphosis and coronal clusters were generally younger and had lower baseline disability, whereas those in the moderate and severe sagittal clusters were older, more disabled, and more likely to have undergone previous spinal surgery.

All four groups experienced significant improvements in health-related quality of life following surgery and achieved broadly similar rates of minimal clinically important difference. However, the magnitude and trajectory of improvement varied between clusters. Patients with predominantly coronal deformity had lower pain and disability scores and better SRS-22 appearance and activity scores at two years. They also achieved the minimal clinically important difference for the SRS-22 appearance domain more frequently than patients with moderate sagittal deformity, at 77.9% versus 58.0%. In contrast, patients with severe sagittal deformity had the greatest residual postoperative disability and the highest rates of major complications and complications leading to reoperation.

These findings illustrate how data-driven clustering can characterise the clinical heterogeneity of adult spinal deformity and identify groups with different postoperative trajectories. However, the clusters were defined primarily by radiographic morphology and should not yet be interpreted as a validated decision-support system for selecting individual treatments. Further prospective and independent validation is required before such approaches can supplement established adult spinal deformity classifications, such as the SRS–Schwab system [44,45].

Contemporary studies are also exploring the use of large language models (LLMs) for image interpretation and decision support. In one of the most recent studies, the ability of several publicly available AI models, including ChatGPT 4 (OpenAI, San Francisco, CA, USA), Microsoft Copilot (Microsoft, Redmond, WA, USA) and Claude (Anthropic, San Francisco, CA, USA) (Opus and Sonnet versions) and others, to classify scoliosis severity based on radiographs and propose optimal treatment was compared with that of a medical model trained on the literature, PMC-LLaMA 13B. Seventy-two radiographs were used, with the Cobb angle measurements performed by two neurosurgeons serving as the reference standard. The results of this experiment indicated that general-purpose language models can, to some extent, assess curvature severity and recommend management, such as observation, bracing, or surgery. However, the specialist model outperformed them in terms of accuracy and consistency of recommendations [46]. The use of LLMs in this field remains experimental, but it represents a promising direction. Ultimately, such models could serve as virtual physician assistants, synthesizing radiological descriptions with specific clinical guidelines.

### 4.4. Machine Learning on Clinical Data: Prediction of Risk and Outcomes

In addition to imaging analysis, machine-learning methods have been investigated for identifying patients at risk of developing scoliosis on the basis of routinely collected clinical data. Vu-Han et al. analysed a longitudinal real-world dataset comprising 84 patients with genetically confirmed 5q-spinal muscular atrophy who had received disease-modifying therapies. The complete dataset contained 1304 clinical visits and more than 360 variables derived from neurological, orthopaedic, respiratory, and functional assessments.

Expert knowledge-guided feature engineering, correlation analysis, and Predictive Power Score analysis were used to identify clinically relevant markers associated with SMA-related scoliosis. These included age, growth-related parameters, HFMSE and 6-min walk test scores, contractures, ventilatory support, orthoses, assistive devices, and gastrostomy. A Random Forest classifier trained using selected clinical and disease-progression features achieved a mean accuracy of 0.82, a precision of 0.85, and an average ROC AUC of 0.87 for identifying scoliosis, defined primarily as a Cobb angle greater than 10° [47].

Patient-grouped cross-validation reduced the risk of data leakage arising from repeated visits by the same individuals. However, the cohort was small and derived from a single specialised centre, and scoliosis labels were based on heterogeneous sources. Dedicated spinal radiographs were available for only 41 patients, while external documentation, chest radiographs, or clinical examinations were used in other cases. Nineteen patients had an unknown scoliosis status and were excluded from modelling. Moreover, no independent external or temporal validation was performed, and calibration was not reported. The findings therefore demonstrate the feasibility of extracting scoliosis-related risk patterns from multidimensional SMA data, but do not yet establish reliable prospective prediction of scoliosis onset or readiness for clinical decision support.

Another potential application of machine learning is the prediction of perioperative outcomes in adult spinal deformity surgery. De la Garza Ramos et al. developed an artificial neural network to predict red blood cell transfusion occurring intraoperatively or within 72 h after surgery. The retrospective analysis used prospectively collected data from 1173 patients included in the 2017–2019 ACS-NSQIP databases. Eighteen patient-related and operative variables were incorporated, including age, sex, ASA class, functional status, body weight, preoperative haematocrit, bleeding disorders, operative duration, pelvic fixation, osteotomy, number of fused levels, and revision status.

The best-performing network contained two hidden layers with 128 and 64 neurons. In the held-out test set, it achieved a sensitivity of 0.80, a positive predictive value of 0.76, an F1 score of 0.78, an accuracy of 0.77, and an AUROC of 0.84 [48]. These findings demonstrate moderate-to-good discrimination and suggest that ANN-based models may help identify patients at increased risk of perioperative transfusion and support blood-product allocation and perioperative risk planning.

However, the model was evaluated using only an internal random train–test split, without independent external or temporal validation. Calibration, specificity, and negative predictive value were not reported. Moreover, some input variables, including actual operative duration, may not be available before surgery, limiting the model’s use as a purely preoperative decision-support tool. The effect of using the model on transfusion rates, complications, resource utilisation, and costs was not prospectively assessed. Consequently, the reported performance should be regarded as evidence of model-development feasibility rather than clinical readiness.

Shi et al. developed an AI-enabled clinical decision-support framework to predict patient-reported and radiographic outcomes after posterior spinal fusion in children with adolescent idiopathic scoliosis. The retrospective analysis used a prospectively maintained multisite cohort of 455 patients treated at two paediatric hospitals. Among the 428 patients with baseline data and at least one postoperative assessment, 378 had 6-month, 351 had 1-year, and 260 had 2-year follow-up data. A total of 171 preoperative features, including demographic characteristics, radiographic measurements, and SRS-22R responses, were used to train five conventional machine-learning models—Gaussian naïve Bayes, logistic regression, Random Forest, support vector machine, and XGBoost—and a multilayer perceptron deep-learning model.

The framework addressed three related tasks: prediction of individual postoperative SRS-22R question responses, achievement of minimal clinically important differences in SRS-22R domains, and a composite outcome combining patient-reported improvement with greater than 60% correction of the major Cobb angle. Among the conventional machine-learning models, Random Forest and support vector machines achieved the highest average AUROCs for individual SRS-22R question prediction, with values of approximately 0.81–0.86 depending on the follow-up horizon. The deep-learning model generally matched or outperformed the conventional models, achieving overall AUROCs of approximately 0.86, 0.84, and 0.83 for predictions at 6 months, 1 year, and 2 years, respectively [49].

Explainability analyses using SHAP and model-specific feature importance identified thoracic apical translation relative to the central sacral vertical line, major coronal Cobb angles, T1 tilt, thoracic kyphosis, lumbar lordosis, sagittal vertical axis, and baseline SRS-22R responses as influential predictors across the different outcome tasks. Radiographic variables were particularly important for predicting improvement in function and pain, whereas baseline patient-reported measures contributed to the prediction of satisfaction and overall SRS-22R improvement. The authors also evaluated model-confidence calibration and gender-related performance differences, representing important steps towards more responsible clinical AI.

However, the study used an internal random patient-level train–test split rather than independent centre-level or temporal validation. The retrospective design, decreasing sample size at longer follow-up, large number of predictors and outcomes, and absence of prospective evaluation of clinical decision impact limit conclusions regarding generalisability and clinical readiness. The findings therefore support the feasibility of personalised outcome prediction and preoperative counselling but do not yet establish that the system improves surgical planning or postoperative rehabilitation outcomes in routine practice.

The methodological characteristics, validation strategies, principal limitations, and current clinical status of AI systems addressing scoliosis screening, deformity classification, automated radiographic measurement, and clinical outcome prediction are summarised in Supplementary Table S2.

## 5. Predictive Models and Machine Learning in Progression and Prognosis

Advances in machine learning (ML) have led to the development of predictive models intended to support prognosis and clinical decision-making in scoliosis. Unlike AI systems focused primarily on automated measurement, these models aim to estimate clinically relevant outcomes, including curve progression, risk of severe deformity, response to conservative treatment, postoperative complications, surgical need, and patient-reported outcomes. By integrating radiological, clinical, anthropometric, biomechanical, and, increasingly, multimodal data, ML-based approaches may help identify patients at higher risk and support more individualized treatment planning. However, compared with automated Cobb angle measurement, predictive modelling remains less clinically mature, as its clinical value depends not only on technical accuracy but also on calibration, external validation, longitudinal follow-up, and evidence that model use improves clinical decision-making in practice.

For prognostic models, discrimination should not be conflated with accurate individual risk estimation. AUC or AUROC describes how well a model ranks patients with and without an outcome, but does not indicate whether predicted probabilities are correct. A model with an AUC of 0.90 may still systematically overestimate or underestimate risk and may perform poorly at the threshold used to intensify follow-up, recommend bracing, prepare blood products, or discuss surgery. Calibration plots, calibration intercept and slope, observed-to-expected ratios, and Brier scores were rarely reported in the reviewed studies. Decision-curve analysis is also needed to determine whether using a model provides greater net benefit than established clinical strategies. In addition, a random train-test split from the same institution is internal validation, not evidence of transportability. Temporal validation using later patients and external validation in different centres, devices, and populations are required to assess dataset shift. When multiple visits or images from the same patient are included, partitioning must occur at the patient level to prevent information leakage.

Classical approaches to estimating the risk of AIS progression were based on a limited number of established clinical and radiographic predictors. One of the most influential examples is the Lonstein–Carlson progression factor, derived from a retrospective cohort of 727 untreated patients with idiopathic scoliosis and initial curve magnitudes ranging from 5° to 29°. Curve progression occurred in 23.2% of patients and was associated with initial curve magnitude and pattern, chronological age, skeletal maturity assessed using the Risser sign, and menarchal status. The final progression factor combined curve magnitude, Risser sign, and chronological age [50]. Although this approach remains historically important, it was not evaluated according to contemporary standards for prognostic modelling. Measures of discrimination, calibration, clinical net benefit, and independent external or temporal validation were not reported, leaving uncertainty regarding the accuracy of individual risk estimates and the model’s transportability to contemporary populations.

More recent machine-learning studies have expanded the range of clinically accessible variables used in scoliosis assessment. Lv et al. developed models based on Random Forest, support vector machine, artificial neural network, decision tree, and generalized linear algorithms using data from 3313 children and adolescents recruited at two hospitals. Seven anthropometric and postural variables were selected: the ratio of sitting height to standing height, lumbar rotation angle, scapular tilt, shoulder-height difference, lumbar concavity, pelvic tilt, and thoracolumbar rotation angle. The artificial neural network achieved the best overall discrimination, with AUCs of 0.899 in the training dataset and 0.897 in the internal validation dataset, and retained the highest performance in a geographically separate validation cohort [51].

However, the model developed by Lv et al. classified the presence of AIS at the time of assessment rather than predicting subsequent curve progression. Its predictors largely represented clinical manifestations of an existing trunk deformity, and radiographic verification was not applied uniformly to all screened participants, creating a potential risk of partial verification bias. Therefore, this model should be interpreted as a non-invasive screening and case-identification tool rather than a prognostic model. Together, these studies illustrate the transition from simple clinically derived progression indices towards more complex machine-learning approaches, while also emphasizing the need to distinguish clearly between detecting existing scoliosis and predicting its future clinical course.

Increasing attention is also being paid to the interpretability of prognostic models. Fang et al. retrospectively analysed 233 patients with adolescent idiopathic scoliosis from three medical centres, each of whom underwent at least three coronal full-spine radiographic examinations. Data from 183 patients treated at one centre were used for model development and internal testing, while 50 patients from two additional centres formed an external validation cohort. Curve progression was defined as an increase of more than 5° in the major Cobb angle between the initial examination and the third follow-up.

Radiographic features were automatically extracted using the VFLDNet keypoint-detection model and used to train six classifiers: logistic regression, support vector machine, decision tree, Random Forest, XGBoost, and K-nearest neighbours. XGBoost achieved the best performance in the external validation cohort, with an AUC of 0.896, an accuracy of 0.88, a sensitivity of 1.00, and a specificity of 0.857 [8]. SHAP analysis identified the change in the major Cobb angle between the first and second follow-ups as the most influential predictor, followed by the major Cobb angle at the second follow-up and the change in the secondary curve angle.

These findings are consistent with clinical reasoning, as early increases in curve magnitude may indicate a greater likelihood of subsequent progression. However, because the model required data from at least two previous radiographic examinations, it should be interpreted as a dynamic model for predicting continued progression rather than as a baseline tool for predicting risk at the initial presentation. Furthermore, the external validation cohort included only 50 patients and eight progression events. The study was retrospective, all centres were located within a single geographic region, and calibration and temporal validation were not reported. Although SHAP improved transparency by showing how individual features influenced model output, it did not establish causal relationships or demonstrate that model-assisted predictions improve follow-up or treatment decisions in clinical practice.

Machine learning has also been investigated for predicting complications following adult spinal deformity surgery. Johnson et al. developed three preoperative artificial intelligence models to predict radiographic proximal junctional kyphosis within two years after surgery. The retrospective single-centre cohort included 191 patients, of whom 89 developed PJK. The first model used a support vector machine incorporating demographic characteristics, planned surgical variables, and conventional coronal and sagittal radiographic measurements. The second combined these structured variables with raw preoperative posteroanterior and lateral scoliosis radiographs, while the third combined them with raw preoperative T1-weighted thoracic MRI.

The clinical and conventional radiographic model achieved a mean sensitivity of 57.2% and specificity of 56.3%. Adding raw scoliosis radiographs modestly improved sensitivity to 68.2%, although specificity remained limited at 58.3%. The best performance was obtained by the MRI-based model, which achieved a mean sensitivity of 73.1% and specificity of 79.5% across the test folds [52]. Attention-map analysis indicated that true-positive predictions were predominantly influenced by posterior thoracic soft-tissue regions, whereas vertebral bodies were not consistently identified as important areas. These findings suggest that information reflecting paraspinal muscle condition and soft-tissue degeneration may contribute to PJK risk prediction beyond conventional alignment measurements.

However, the MRI analysis was based on a subgroup of only 59 patients. The study was retrospective, conducted at a single institution, and relied exclusively on internal nested cross-validation without independent external or temporal validation. Calibration and the clinical consequences of false-positive and false-negative predictions were not reported. Moreover, the attention maps identify image regions associated with model predictions but do not establish a causal relationship between muscle degeneration and PJK. The findings therefore support the potential value of preoperative soft-tissue imaging but require confirmation in larger, prospectively recruited multicentre cohorts before the model can inform surgical planning or preventive interventions.

A similar exploratory approach was applied to the analysis of outcomes following conservative treatment. Ayvaz et al. retrospectively examined 128 individuals who had undergone Schroth-based exercise therapy for periods ranging from 3 to 36 months. Random Forest regression models using age, baseline Cobb angle, and treatment duration were developed to predict Cobb-angle change and pain reduction. Baseline curve magnitude was the most influential feature for predicting Cobb-angle change, followed by age, whereas treatment duration had a smaller contribution. In the pain-reduction model, treatment duration was the most influential feature, and the model achieved a reported MAE of 0.84 and RMSE of 1.06 [53].

The study also evaluated an active-learning strategy, but this analysis addressed classification of scoliosis direction rather than prediction of treatment response. Classification accuracy increased from 65% to 85% across five simulated labeling iterations, suggesting that uncertainty-based sampling may reduce labeling requirements in small clinical datasets. However, this result should not be interpreted as evidence that active learning improved therapeutic outcomes or enabled selection of patients for longer treatment.

Several methodological and reporting issues substantially limit interpretation. Although the study was presented as an analysis of adolescent idiopathic scoliosis, approximately half of the cohort consisted of adults, including individuals aged 70 years or older, and the reported median baseline Cobb angle was below the conventional diagnostic threshold for scoliosis. The retrospective uncontrolled design, exclusion of patients with poor adherence, heterogeneous treatment duration, unclear model-validation procedures, absence of calibration and external validation, and inconsistencies in the reported Cobb-angle and economic results preclude causal conclusions. The study should therefore be considered exploratory evidence that machine-learning methods may identify nonlinear associations in conservative-treatment data, rather than validation of a clinically deployable model or proof that ML-guided Schroth therapy improves outcomes or is cost-effective.

Recent studies in scoliosis-related and broader spine surgery also demonstrate a shift from predominantly radiographic endpoints towards operational and patient-centred outcomes. Park et al. evaluated retrieval-augmented prediction for forecasting postoperative length of stay using structured electronic health-record data and operative notes from spine surgery cases. The best-performing approach combined a neural network with similarity-based retrieval and achieved an R^2^ of 0.52 and an MAE of 4.16 days [54]. Although this study was not specific to scoliosis or spinal deformity, it illustrates the potential use of predictive models for perioperative resource planning.

Patient-reported improvement has become another important predictive target. Hosozawa et al. compared TabNet, a deep neural network, and elastic-net penalised logistic regression for predicting clinically important improvement after lumbar spine surgery. The models were developed using data from 981 patients from two centres and externally validated in 168 patients from a third centre. Mean AUCs in the external cohort ranged from approximately 0.77 to 0.80 across JOABPEQ and pain outcomes, with no clear advantage of deep learning over penalised regression [55]. André et al. prospectively evaluated a previously developed deep-learning model in 103 patients undergoing lumbar decompression surgery. The model achieved an AUC of 0.816, sensitivity of 81%, and specificity of 59% for predicting a composite clinically important postoperative improvement [56]. However, the relatively small validation cohort, moderate specificity, and previous use of synthetically generated records during model development limit conclusions regarding transportability and routine clinical use. Both studies relate to broader lumbar spine surgery rather than specifically to scoliosis and should therefore be interpreted as contextual evidence.

More directly relevant evidence was provided by Vila et al., who examined prediction of self-image satisfaction after adult spinal deformity surgery. Their retrospective analysis of a prospective multicentre registry included 710 surgically treated patients with a minimum two-year follow-up. Random Forest models were developed separately for three previously validated deformity phenotypes and predicted achievement of the patient-acceptable symptom state in the SRS-22 self-image domain, with AUCs ranging from 0.73 to 0.94 depending on the phenotype. Functional disability, pain, and physical and social functioning were more influential predictors than radiographic alignment parameters or postoperative complications [57]. These findings illustrate a clinically important movement away from exclusively radiograph-centred definitions of success towards outcomes that more directly reflect the patient’s experience.

Overall, predictive modelling in scoliosis and related spinal deformities has demonstrated feasibility across several clinically relevant tasks, including estimation of curve progression, postoperative complications, response to conservative treatment, length of hospital stay, and patient-reported improvement. Nevertheless, performance remains heterogeneous, and direct comparison between studies is difficult because of differences in populations, outcomes, prediction horizons, feature definitions, and validation strategies. Several models achieved moderate-to-good discrimination, but high AUC values should not be interpreted as evidence of accurate individual risk estimation or clinical effectiveness.

The principal limitation remains the gap between model development and clinical implementation. Many studies were retrospective, used relatively small or selected cohorts, or relied primarily on internal cross-validation. Even where external or prospective validation was performed, calibration, decision-curve analysis, temporal transportability, subgroup performance, and the consequences of false-positive and false-negative predictions were often incompletely assessed. Most importantly, none of the reviewed models demonstrated through a prospective impact study that providing AI-generated predictions to clinicians improved treatment selection, follow-up strategies, resource allocation, complication rates, or patient outcomes.

Explainability methods such as SHAP and Grad-CAM may improve model transparency by indicating which variables or image regions contributed to individual predictions. However, feature importance and attention maps do not establish causal relationships and may themselves be unstable across datasets. Explanations should therefore be treated as tools for model auditing and hypothesis generation rather than as independent evidence of biological mechanisms.

Future studies should prioritise prospectively defined prediction horizons, patient-level data partitioning, independent geographical and temporal validation, comprehensive calibration assessment, and decision-curve analysis at clinically relevant thresholds. Multimodal and longitudinal models integrating radiographic, clinical, functional, biomechanical, and patient-reported data may improve prediction, but the added complexity must be justified through transparent reporting, reproducibility, and demonstrable clinical benefit. Ultimately, the value of predictive artificial intelligence in scoliosis will depend not on statistical performance alone, but on whether its use leads to safer, more consistent, and more patient-centred clinical decisions.

The methodological characteristics, validation strategies, principal limitations, and current clinical readiness of prognostic and outcome-prediction models in scoliosis and related spinal disorders are summarised in Supplementary Table S3.

## 6. AI in Surgical and Postoperative Treatment

Artificial intelligence is increasingly being investigated as a tool for supporting scoliosis surgery, particularly in preoperative deformity classification, fusion-level selection, simulation of postoperative spinal alignment, and prediction of mechanical complications. These applications differ from automated radiographic measurement because their intended purpose is not merely to quantify deformity, but to provide information that may influence operative strategy or postoperative risk assessment. Nevertheless, most available systems remain at the development or retrospective validation stage, and evidence that their use improves surgical decisions or patient outcomes remains limited.

Accurate Lenke classification is an important component of adolescent idiopathic scoliosis surgical planning because it incorporates curve structure, lumbar modifiers, and sagittal alignment. Dong et al. developed a multiple-view deep-learning system based on U-Net semantic segmentation that automatically aligned standing anteroposterior, lateral, and side-bending radiographs, measured Cobb angles, and assigned Lenke classifications. The model was developed using 1812 radiographs from 506 patients and evaluated prospectively in an additional 107 cases. The automated system demonstrated higher reported consistency than manual classification for curve type, lumbar modifier, and sagittal modifier, with coefficients of 0.989, 0.932, and 0.987, respectively [58]. However, the study primarily evaluated agreement with manual classification and did not assess whether automated classifications led to appropriate fusion-level selection or improved postoperative outcomes.

A more recent multistage system developed by Xu et al. combined Swin-Unet vertebral segmentation, DeepLabv3+ localisation of lumbar pedicles, automated Cobb-angle measurement, and a feature-fusion module for end-to-end Lenke classification. The retrospective dataset included 650 individuals, including 467 patients with AIS and 183 controls. In an independent test set, the model achieved an overall Lenke-classification accuracy of 95.6% and a macro-averaged F1 score of 0.862. Agreement between automated and expert Cobb-angle measurements was also high, with ICCs ranging from 0.969 to 0.976 [59]. Although the interpretable, modular architecture represents a promising step towards standardised preoperative assessment, the system was not independently validated in a geographically separate institution and did not directly generate a complete surgical strategy.

Machine learning has also been used to accelerate biomechanical simulation of surgical correction. Phellan Aro et al. trained surrogate ML models using 6400 finite-element simulations generated from the three-dimensional spinal geometries of 64 patients with AIS. Principal component analysis followed by linear regression produced estimates of postoperative vertebral position with an average error of 3.75 mm and orientation errors below 2.74° in all three principal axes. Predictions were generated within approximately 3 ms, compared with seconds or minutes for conventional finite-element simulations [60]. This approach may enable surgeons to interactively compare several hypothetical correction targets during preoperative planning. However, the model reproduced outputs generated by finite-element simulations rather than actual postoperative results. Consequently, its accuracy depends on the assumptions and validity of the underlying biomechanical model, and prospective comparison with observed postoperative anatomy is still required.

He et al. developed ScoliosisPLAN, an integrated artificial intelligence system designed to combine preoperative fusion-level planning with prediction of postoperative coronal alignment in adolescent idiopathic scoliosis. The system consisted of two complementary modules. ScolioPlanNet, derived from the YOLOv8 instance-segmentation architecture, used preoperative standing and bending radiographs to predict the upper and lower instrumented vertebrae and the total number of fused segments. ScolioPredNet used a latent diffusion model to generate a simulated postoperative radiograph conditional on the proposed fusion strategy.

The multicentre study included 1425 surgically treated patients with AIS and at least two years of postoperative follow-up. A retrospective cohort of 1143 patients from one institution was used for model training and validation, while performance was evaluated in a prospectively recruited internal cohort of 200 patients and external prospective cohorts of 38 and 44 patients from two additional institutions. In the internal prospective test set, ScolioPlanNet achieved a mean absolute error of 0.34 vertebral levels for the total fusion length, compared with 0.30 for a surgeon with 14 years of experience and 0.67 for a surgeon with 5 years of experience. In the two external cohorts, the corresponding errors were 0.63 and 0.52 vertebral levels, indicating reasonable transportability across institutions [61].

ScolioPredNet also predicted clinically relevant postoperative radiographic outcomes. In the internal prospective cohort, the mean absolute errors were 2.94° for the postoperative major Cobb angle, 2.60° for the secondary Cobb angle, and 2.92 mm for coronal balance. Performance remained comparatively stable in the external cohorts despite differences in patient characteristics and imaging equipment. The integration of spinal-flexibility information from bending radiographs consistently improved fusion-planning performance, supporting the clinical relevance of incorporating deformity flexibility rather than relying on standing radiographs alone.

However, ScoliosisPLAN reproduced fusion strategies previously selected by surgeons rather than identifying an independently established optimal surgical construct. The implemented fusion levels therefore served as the reference standard, and agreement with these decisions does not demonstrate that the proposed plan preserves the maximum number of mobile segments or minimises long-term complications. Furthermore, 42 patients who developed long-term coronal complications were excluded from the primary fusion-planning performance analysis, although the model agreed with the original surgical plan in 28 of these cases. This finding illustrates that learning prevailing expert practice may also reproduce limitations inherent in existing decision-making. ScoliosisPLAN should therefore be considered an advanced decision-support and outcome-simulation system rather than an autonomous surgical-planning tool. Prospective impact studies comparing AI-assisted planning with standard multidisciplinary planning are required to determine whether its use improves fusion-level selection, postoperative alignment, complication rates, revision surgery, and patient-reported outcomes.

Large language models have also been evaluated for fusion-level planning, although their performance appears highly dependent on the type of input and task structure. Jia et al. provided DeepSeek-R1 with structured clinical and radiological information from 203 consecutive patients with AIS who met surgical indications. Three experienced spine surgeons considered the proposed upper and lower instrumented vertebrae surgically reasonable in 70.9% of cases. Performance was relatively high for Lenke types 1A, 1B, 5C, and 6C, but substantially lower for types 1C, 2A, and 2C, for which acceptable recommendations were generated in only 19.0%, 42.9%, and 25.0% of cases, respectively [62]. The study therefore demonstrates that reasoning-oriented LLMs may produce plausible recommendations in relatively straightforward curve patterns, but their performance is not sufficiently consistent for autonomous planning. Moreover, the reference standard was based on surgeons’ ratings of reasonableness rather than on comparison with actual operative plans and long-term outcomes.

Considerably poorer results were obtained when general-purpose multimodal LLMs were required to interpret radiographic images directly. Aktan et al. evaluated ChatGPT, Claude, Gemini, and DeepSeek using standing and side-bending radiographs from 125 surgically treated patients with AIS. Agreement with expert consensus ranged from only 1.6% to 10.2% for Lenke classification and from 0.8% to 12.0% for fusion-level selection. Test–retest reliability was close to chance despite identical radiographs and prompts, and incorrect end-vertebra selection occurred in approximately 74–81% of assignments [63]. These findings show that rapid output generation should not be confused with clinically reliable reasoning. They also suggest that structured language-based analysis of pre-extracted measurements and direct multimodal interpretation of raw radiographs represent fundamentally different tasks and should not be compared without considering the input format and reference standard.

Artificial intelligence has also been applied to the assessment of postoperative mechanical-complication risk in adult deformity surgery. Lan et al. developed a deep-learning system for vertebral detection and automated calculation of several versions of the Global Alignment and Proportion score using 3485 radiographs with and without spinal instrumentation. The system automatically extracted sagittal parameters and generated editable GAP-score variants. An ethnicity-, age-, and sex-adjusted version achieved the highest discrimination for mechanical complications after adult degenerative scoliosis surgery, although its AUC was only 0.627 [64]. This modest discrimination indicates that automation may improve workflow efficiency and measurement reproducibility without necessarily producing a sufficiently accurate prognostic model. External validation, calibration analysis, and evaluation at clinically meaningful intervention thresholds remain necessary.

The limitations of relying exclusively on spinal alignment were further demonstrated by Sundrani et al. in a retrospective cohort of 231 patients undergoing adult spinal deformity surgery, of whom 147 developed at least one mechanical complication. Seven machine-learning models were developed using different combinations of alignment, bone-quality, and paraspinal soft-tissue variables. The model combining all three domains achieved a positive predictive value of 0.90, sensitivity of 0.67, and specificity of 0.84, outperforming models based on alignment alone [65]. These findings support the concept that mechanical complications are influenced by the interaction between correction strategy, bone quality, and soft-tissue condition. However, the analysis incorporated postoperative alignment measurements, limiting its interpretation as a purely preoperative risk-prediction tool. The high prevalence of complications in the cohort also affects the reported positive predictive value, which may not transfer directly to centres with different case selection or complication rates.

Overall, AI applications directly relevant to scoliosis surgery are progressing from automated description of deformity towards decision-support tasks such as Lenke classification, fusion-level selection, biomechanical simulation, and prediction of postoperative mechanical failure. The available evidence nevertheless remains heterogeneous. Some specialised computer-vision systems achieve high agreement with expert classification, whereas general-purpose multimodal language models remain unreliable when required to interpret complex spinal radiographs or recommend fusion levels. Similarly, biomechanical surrogate models can produce rapid simulations, but their outputs have not yet been prospectively validated against actual postoperative anatomy.

Before these systems can influence operative treatment, future studies should use independent multicentre and temporal validation, clearly defined patient-level data partitioning, comprehensive calibration analysis, and clinically relevant decision thresholds. Model outputs should also be compared with actual operative plans, postoperative alignment, complications, revision rates, and patient-reported outcomes rather than solely with retrospective expert annotations. Prospective clinical-impact studies are required to determine whether AI-assisted planning improves consistency, preserves motion segments, reduces complications, or provides measurable benefit over multidisciplinary expert assessment. At present, AI may support scoliosis surgeons by standardising measurements and presenting additional risk information, but it should not independently determine deformity classification, fusion levels, or the extent of correction.

The methodological characteristics, validation strategies, principal limitations, and current clinical readiness of AI systems developed for scoliosis surgical planning and postoperative care are summarised in Supplementary Table S4.

## 7. Language Models as Auxiliary Tools in Education, Communication, and Research

Language-based artificial intelligence models are increasingly being investigated as auxiliary tools for patient education, communication, and information processing in scoliosis care. Their most clinically plausible near-term applications include simplifying written educational materials, answering frequently asked patient questions, summarising information, and identifying recurring patient concerns. These functions should be distinguished from diagnostic interpretation or therapeutic decision-making, for which general-purpose language and multimodal models remain substantially less reliable.

One potential application is the improvement of health-information readability. Kirchner et al. used a generative conversational model to simplify educational materials concerning scoliosis and other orthopaedic conditions. For scoliosis-specific materials, the median Flesch–Kincaid grade level decreased from 12.6 to 5.6 after AI-assisted rewriting. The converted texts retained sufficient detail for educational use, and the investigators identified no factual inaccuracies [37]. However, the study evaluated linguistic readability and expert-reviewed content preservation rather than actual patient comprehension, recall, treatment adherence, or shared decision-making. It therefore demonstrated that AI can simplify existing clinician-curated materials, but not that AI-generated education improves clinical outcomes.

More recent findings indicate that readability depends strongly on the selected model and generation strategy. Zhao et al. compared five language models using questions concerning adolescent idiopathic, congenital, and neuromuscular scoliosis. DeepSeek-R1 produced the most accessible response, with a Flesch–Kincaid grade level of 6.2, whereas selected outputs from ChatGPT-o1 and ChatGPT-o3 mini-high exceeded the 12th-grade level. Despite these substantial readability differences, all five systems achieved similar DISCERN scores of approximately 50 out of 80, corresponding to only fair informational quality, and none provided verifiable citations [11]. These results suggest that fluent or simplified language should not be interpreted as evidence of completeness, reliability, or source transparency.

Large language models have also been evaluated as sources of responses to scoliosis-related frequently asked questions. Suresh et al. submitted 31 questions concerning operative and non-operative scoliosis care to ChatGPT, Google Gemini, and Microsoft Copilot. Four fellowship-trained orthopaedic surgeons rated ChatGPT highest, with a mean score of 4.0 out of 5, compared with 3.1 for both Gemini and Copilot [66]. Nevertheless, the evaluation involved only four raters from one institution, model versions were not clearly documented, and the study assessed perceived answer quality rather than concordance with predefined clinical guidelines.

Other studies have demonstrated more variable performance. Lang et al. evaluated ChatGPT-3.5, ChatGPT-4, and Google Bard responses to ten frequently asked AIS questions using a blinded group of experienced deformity surgeons from seven European spine centres. Only 26% of all responses were rated as excellent, while 22% contained clear mistakes and 9% were off-topic. ChatGPT-4 achieved the most favourable ratings, but significant disagreement between evaluators highlighted the subjectivity of expert-based assessment [35]. Giray et al. subsequently evaluated ChatGPT-3.5 using 64 questions spanning general information, diagnosis, treatment, follow-up, and quality of life. Although 53.1% of responses were considered correct and comprehensive and a further 34.4% correct but incomplete, only 26.3% of treatment and follow-up responses were fully correct. Repeated answers were consistent in 76.6% of cases [67]. These results indicate that general educational explanations may be acceptable, whereas recommendations involving treatment selection and follow-up require greater caution.

Comparisons with internet search results and between different model generations have produced similarly heterogeneous findings. Tekin et al. found that ChatGPT responses to ten commonly searched scoliosis questions received more favourable expert ratings than answers presented through Google search [68]. However, comparing a conversationally generated response with search-engine excerpts does not constitute an equivalent comparison between information systems. Vu-Han et al., assessing ten frequently asked questions, found no statistically detectable difference between GPT-3.5 and GPT-4.0, with responses generally requiring minimal or moderate clarification; the small question set and limited inter-rater reliability prevented conclusions regarding model equivalence [69].

Newer models have not eliminated these limitations. Aydin et al. evaluated Claude Sonnet 4.5 and GPT-5.2 using 100 patient-oriented AIS questions covering ten clinical domains. Both models achieved 91% factual accuracy. However, only approximately one third of responses were considered sufficiently clear and understandable, while adequate conceptual coverage was achieved in 46% of Claude responses and 29% of GPT responses [70]. This discrepancy between factual correctness, clarity, and conceptual completeness is clinically important: an answer may contain no explicit false statement while still omitting information required for informed decision-making.

Overall, available FAQ studies remain cross-sectional benchmark evaluations rather than clinical implementation studies. Responses were generally assessed by clinicians using investigator-developed rating scales, with limited participation from adolescents, parents, or individuals with different health-literacy levels. The studies did not determine whether patients understood the information, made better-informed decisions, experienced less anxiety, adhered more closely to bracing or rehabilitation, or communicated more effectively with clinicians. Their results are also time-sensitive because model versions, system instructions, retrieval mechanisms, safety policies, and generated responses may change without transparent documentation.

Natural language processing may additionally provide insight into patient experiences and unmet communication needs. Dominy et al. analysed publicly available scoliosis surgery-related posts from Twitter, Instagram, and Reddit. Positive posts frequently described postoperative progress, satisfaction, and educational experiences, whereas negative discussions concerned waiting times, persistent pain, preoperative fear, and uncertainty about surgical risks [71]. However, social-media users constitute a self-selected population, and platform-specific posting patterns cannot be considered representative of all patients undergoing scoliosis surgery. Such analyses are therefore better suited to identifying topics for counselling and hypothesis generation than to estimating the prevalence of patient concerns.

As discussed in the preceding sections, favourable performance in educational question-answering should not be extrapolated to diagnostic or therapeutic tasks. General-purpose language and multimodal models have shown poor reliability in direct radiographic interpretation, quantitative Cobb-angle measurement, Lenke classification, fusion-level selection, and individualised treatment planning. These higher-risk applications require task-specific development, reproducible image processing, external validation, and comparison with actual clinical outcomes rather than reliance on persuasive natural-language output.

Several additional limitations affect the reproducibility of LLM studies. Model names alone may not identify the exact system version, knowledge state, safety configuration, or retrieval functionality used at the time of testing. Outputs may also vary according to prompt wording, chat history, sampling parameters, language, and repeated querying. Consequently, future studies should report the complete prompt, date of access, model version, system configuration, response-generation procedure, number of repeated runs, source-verification method, and inter-rater agreement. Assessments should distinguish factual correctness, completeness, readability, empathy, guideline concordance, citation accuracy, stability, and potential clinical harm rather than combining them into a single subjective quality score.

Generative AI may also support research ideation. Leonardo et al. asked ChatGPT-3.5, Gemini-1.5, Copilot, and Llama-3 to propose research ideas concerning AIS and assessed whether similar topics could be identified through modified keyword searches in PubMed. Research ideas without an identified PubMed match represented 16.7% of ChatGPT suggestions, 10.0% of Copilot suggestions, 8.3% of Llama suggestions, and none of the Gemini suggestions [72]. However, absence of a result in a simplified PubMed search does not establish scientific novelty. The study used a single broad prompt, a small number of generated ideas, and no formal expert assessment of feasibility, methodological quality, clinical importance, or overlap with research outside PubMed. Such models may therefore assist brainstorming, but their outputs should be treated as preliminary hypotheses requiring comprehensive literature review and expert evaluation rather than as evidence of a genuine research gap.

Overall, current evidence supports a limited, task-specific, and clinician-supervised role for language-based AI in scoliosis care. Its strongest near-term use is as a tool for rewriting clinician-approved information, generating preliminary educational drafts, summarising material, and identifying questions that should be addressed during consultations. Even in these lower-risk applications, outputs require verification for accuracy, completeness, readability, and source traceability. Diagnostic interpretation, treatment recommendations, and surgical planning should remain clinician-led. Future studies should move beyond expert ratings of isolated outputs and assess whether carefully governed AI-assisted communication measurably improves patient comprehension, shared decision-making, adherence, satisfaction, and clinical outcomes.

### Limitations and Future Directions

Despite rapid progress, the clinical translation of AI in scoliosis diagnosis and management remains limited by several methodological and practical challenges. First, many models have been developed and validated on relatively small, homogeneous, or single-centre datasets, often acquired under controlled conditions. This raises concerns about generalizability across different populations, imaging systems, acquisition protocols, and clinical environments. Models trained on EOS images, conventional radiographs, surface topography, or standardized back photographs may not perform equally well when applied to different devices, image qualities, patient body habitus, or home-based photographs. Atypical cases, including very severe deformities, postoperative images, poor-quality scans, and patients with unusual skeletal abnormalities, also remain challenging. Therefore, prospective multicentre validation using diverse datasets is essential before these systems can be reliably implemented in routine practice.

A second important limitation is that many AI tools address narrow tasks, such as binary scoliosis detection, Cobb angle measurement, coronal-plane analysis, curve classification, or prediction of a specific outcome. Although these applications can improve speed, reproducibility, and screening sensitivity, they do not yet replace comprehensive clinical assessment, particularly in complex three-dimensional deformities, treatment planning, fusion-level selection, or long-term outcome prediction. The “black box” nature of many deep learning models also remains a barrier to clinician trust. Explainable AI methods, such as attention maps, Grad-CAM visualizations, feature-importance analysis, and uncertainty estimation, should therefore become standard components of future models, especially when AI outputs influence clinical decision-making.

Beyond technical performance, the widespread clinical implementation of AI in scoliosis care will depend on overcoming several regulatory, ethical, and organizational barriers. Systems intended to influence diagnosis, risk stratification, or treatment planning must undergo regulatory evaluation as medical devices, with a clearly defined intended use, reproducible validation, transparent documentation of model development, and evidence of safety and clinical benefit. Particular challenges arise for continuously updated or adaptive algorithms, which require mechanisms for version control, performance auditing, detection of dataset shift, and post-deployment surveillance. Data protection is equally important because the development of these systems frequently relies on large collections of radiographs, clinical records, surface images, and, increasingly, cloud-based or mobile data. Robust de-identification, secure storage and transfer, controlled access, and appropriate governance of multicentre data sharing are therefore essential to protect patient confidentiality. Clinical acceptance will also depend on explainability and accountability. AI outputs should be accompanied by clinically meaningful information, such as identified anatomical landmarks, uncertainty estimates, contributing variables, and warnings when an input falls outside the model’s validated scope. Models should also be evaluated across sex, age, ethnicity, curve severity, body habitus, and other clinically relevant subgroups to identify performance disparities and reduce the risk of unequal quality of care. Finally, implementation should be designed around existing clinical workflows rather than requiring clinicians to use separate research platforms. Integration with PACS, electronic health records, radiology reporting systems, and surgical planning software should minimize additional workload, support clinician review and correction, and preserve clear human oversight. Prospective implementation studies should therefore evaluate not only diagnostic accuracy, but also usability, interoperability, time efficiency, error management, clinician acceptance, patient outcomes, and the allocation of responsibility when AI-supported recommendations prove incorrect.

Most systems discussed in this review were evaluated as research prototypes, and the reviewed publications generally did not report FDA clearance, CE marking, NMPA approval, or other regulatory authorization for scoliosis-specific clinical use. Consequently, favourable performance in retrospective, prospective, or external validation studies should not be interpreted as equivalent to regulatory approval or readiness for routine deployment. Regulatory requirements will depend on the system’s intended purpose, degree of autonomy, target population, and potential influence on diagnostic or therapeutic decisions. Future studies should therefore clearly distinguish research-only systems from authorized medical devices and report the jurisdiction, intended use, and scope of any applicable regulatory approval.

Evidence regarding the economic impact of AI implementation in scoliosis care also remains limited. Potential savings resulting from faster measurements, reduced clinician workload, improved reproducibility, and earlier detection must be balanced against the costs of software licensing, computing infrastructure, integration with hospital systems, staff training, cybersecurity, maintenance, performance monitoring, and regulatory compliance. Screening systems with high sensitivity but limited specificity may additionally increase downstream expenditure through unnecessary radiographs, specialist referrals, repeated assessments, and investigation of false-positive findings. Conversely, false-negative results may delay diagnosis or treatment and generate further clinical and economic consequences. Future implementation studies should therefore include cost-effectiveness and budget-impact analyses alongside measures of diagnostic performance, workflow efficiency, and patient outcomes.

Future research will likely move toward multimodal and clinically interpretable systems that combine radiographs, surface images, clinical measurements, biomechanical data, genetic information, and patient-reported outcomes. Generative AI may support dataset expansion, radiation-reduction strategies, and synthetic image generation, but its outputs require careful validation before clinical use. Similarly, large language models may assist with patient education, communication, information summarization, and clinical documentation, although physician oversight remains necessary. Overall, AI has the potential to improve the precision, efficiency, and personalization of scoliosis care; however, robust external validation, transparent reporting, explainable design, regulatory authorization, economic evaluation, and prospective outcome-based studies are required before these technologies can be widely and safely translated into clinical practice.

## Data Availability

No new data were created or analyzed in this study. Data sharing is not applicable to this article.

## Supplementary Materials

The following supporting information can be downloaded at: https://www.mdpi.com/article/10.3390/jcm15166361/s1, Table S1: Methodological characteristics, validation strategies, performance, and clinical readiness of artificial intelligence systems for scoliosis imaging and automated assessment. Table S2: Methodological characteristics, validation strategies, performance, and clinical readiness of AI systems for scoliosis screening, deformity classification, automated radiographic measurement, and outcome prediction. Table S3: Methodological characteristics, validation strategies, performance, and clinical readiness of predictive models in scoliosis and related spinal disorders. Table S4: Methodological characteristics, validation strategies, performance, and clinical readiness of artificial intelligence systems for scoliosis surgical planning and postoperative care.

## Abbreviations

The following abbreviations are used in this manuscript:

6MWT	6-min walk test
AI	artificial intelligence
AIS	adolescent idiopathic scoliosis
AL	active learning
ANN	artificial neural network
AP	anteroposterior
ASD	adult spinal deformity
AUC	area under the curve
AUROC	area under the receiver operating characteristic curve
CLIP	contrastive language–image pre-training
CNN	convolutional neural network
CT	computed tomography
DASPP	dense atrous spatial pyramid pooling
EOS	EOS biplanar imaging system
FAQ	frequently asked questions
GAN	generative adversarial network
Grad-CAM	gradient-weighted class activation mapping
HFMSE	Hammersmith Functional Motor Scale Expanded
ICC	intraclass correlation coefficient
LLM	large language model
LSTM	long short-term memory
MAE	mean absolute error
MCID	minimal clinically important difference
ML	machine learning
MRI	magnetic resonance imaging
NLP	natural language processing
NURBS	non-uniform rational B-splines
PACS	picture archiving and communication system
PCK	percentage of correct keypoints
PJK	proximal junctional kyphosis
ROC	receiver operating characteristic
ROSHTSH	ratio of sitting height to standing height
SHAP	Shapley additive explanations
SMA	spinal muscular atrophy
SRS-22	Scoliosis Research Society-22 questionnaire
SRS-22R	Scoliosis Research Society-22 revised questionnaire
SVA	sagittal vertical axis
SVM	support vector machine
VAS	visual analogue scale
