# Supplementary material for: From Algorithms to Clinics: Recent Progress in AI for Scoliosis Diagnosis and Management
Source: J Clin Med. 2026 Aug 18;15(16):6361. doi: 10.3390/jcm15166361 (PMC13513889; doi:10.3390/jcm15166361)
Supplement: Supplementary file 1 [file jcm-15-06361-s001.zip › Supplementary Table S1.pdf]

Supplementary Table S1. Methodological characteristics, validation strategies, performance, and clinical readiness of artificial intelligence systems for scoliosis imaging and automated assessment

Study	Clinical task, input, and AI approach	Dataset and validation strategy	Principal reported performance	Structured methodological appraisal	Clinical translation and readiness
**Yang et al. [17]**	Scoliosis detection and severity grading from unclothed back photographs; Faster R-CNN for back localisation and ResNet-based classification	3,240 patients for development and internal validation; 400-patient external validation dataset; data obtained from three institutions; fivefold cross-validation	Internal AUC 0.946, sensitivity 87.5%, and specificity 83.5% for scoliosis ≥10°. In external validation, AUC was 0.811, sensitivity 80.7%, and specificity 58.0%. For curves ≥20°, external AUC was 0.929	Large development cohort and separate external dataset. Performance decreased during external testing, particularly specificity and four-class severity accuracy. Population restricted to Chinese adolescents. Calibration and temporal validation were not reported	Cloud-based self-screening platform was developed. Potential to reduce unnecessary referrals was discussed, but prospective workflow, regulatory status, and formal health-economic outcomes were not evaluated. **Readiness: external diagnostic validation**
**Mohamed et al. [19]**	Radiation-free screening using three-dimensional markerless surface topography and	Dataset size and data partitioning require confirmation from the full text; no independent	Accuracy approximately 95%, sensitivity 97%, and specificity 90% for binary scoliosis	Promising screening discrimination, but binary classification does not quantify curve	Requires dedicated acquisition technology and broader community-based

	CNN-based trunk-asymmetry classification	external or temporal validation is described in the current draft	detection	magnitude or identify clinically relevant treatment thresholds. Calibration, subgroup performance, and failure analysis were not reported	validation. Workflow integration, regulatory status, and economic impact were not evaluated. **Readiness: proof of concept/internal validation**
**Duan et al. [10]**	Bare-back image segmentation and scoliosis severity classification; Dual Attention U-Net with a computer-vision-derived asymmetry index	458 participants from three hospitals: 321 training, 46 validation, and 91 testing; random pooled split rather than hospital-level external validation	AUC 0.93 for scoliosis detection; AUC 0.95 for severe scoliosis; overall four-class accuracy 86.9%; severe-category accuracy, precision, and recall above 90%	Explicit training, validation, and test sets and specialist-derived radiographic reference standard. However, image acquisition was standardised, low-quality images were excluded, and no centre-held-out or temporal validation was performed. Calibration was not assessed	Processing was faster than visual assessment, but real-world performance with uncontrolled photographs remains uncertain. No regulatory, workflow-integration, or formal cost-effectiveness evidence. **Readiness: internal diagnostic validation**
**Zhang et al. [20]**	Smartphone-	2,158 patients	Sensitivity 84.9%	Prospective	Direct mobile-app

	based classification of AIS severity, curve type, and progression; ScolioNets incorporated into the AlignProCARE application	overall; 1,780 in model development and 378 in a prospective validation cohort; radiographic assessment by specialists used as the reference standard	and NPV 89.2% for identifying patients requiring observation; sensitivity 82.6% and NPV 90.0% for identifying patients requiring consideration of surgery	validation and clinically relevant triage categories are important strengths. However, external multicentre and temporal validation were not demonstrated, and performance may be affected by device, positioning, background, and body habitus. Probability calibration was not reported	workflow provides plausible out-of-hospital integration. False-negative results could delay radiographic assessment or specialist referral. Regulatory status and downstream cost consequences were not evaluated. **Readiness: prospective diagnostic validation**
**Jiang et al. [21]**	Segmentation of bony structures on two-dimensional spinal ultrasound; ResNet-101-based UGBNet with spatial/channel attention and multiscale feature extraction	Relatively small experimental dataset; exact patient and image counts should be confirmed from the full text; internal model comparison without external validation	Dice score 74.2%, outperforming conventional U-Net and other comparison architectures	Appropriate segmentation metric was reported, but the study did not validate Cobb angle measurement, clinical classification, progression monitoring, or	May support radiation-free three-dimensional visualisation, but clinical benefit remains indirect because segmentation accuracy was not linked to patient-management decisions. No

				performance across centres and devices. No temporal validation or clinically defined failure threshold	regulatory, workflow, or economic evaluation. **Readiness: technical proof of concept**
**Harake et al. [18]**	Automated prediction of sagittal spinopelvic parameters from whole-spine radiographs; SpinePose deep-learning model	761 radiographs for model training and validation; separate test set of 40 radiographs assessed by four specialist reviewers	ICCs 0.91–1.00; median errors included 2.2 mm for SVA and approximately 1.1°–2.6° for angular parameters	Specialist comparison and inclusion of heterogeneous anatomy and instrumentation strengthen applicability. The independent test set was small, and no external-centre or temporal validation was reported. Agreement was emphasised more than failure rates or threshold-crossing errors	Potentially useful for surgical planning and patient selection, but prospective effects on decisions, workflow time, outcomes, and costs remain untested. Regulatory status was not reported. **Readiness: advanced internal validation**
**Xie et al. [22]**	Automated coronal and sagittal measurement and severity	600 paired radiographic examinations from one centre; random 3:1	Coronal MAD 2.15° and ICC 0.985; sagittal MAD 2.72° and ICC 0.927; diagnostic	Simultaneous coronal and sagittal assessment is a strength.	Misclassification of severe curves could delay specialist referral or influence

	classification from paired AP and lateral radiographs; ResNet34 with U-Net-derived features	training–test split	accuracy 97.2%; severity accuracy 94.5%; sensitivity 88.2% for severe curves >45°	Retrospective single-centre design, internal random split, and exclusion of poor-quality, postoperative, brace, and severe-pelvic-tilt images limit generalisability. Approximately 12% of severe cases were not classified as severe. Calibration and temporal validation were not reported	treatment planning, supporting assistive rather than autonomous use. No PACS integration, regulatory status, or economic analysis was provided. **Readiness: internal clinical validation**
**Chen et al. [23]**	Fully automated Cobb angle and end-vertebra selection on AP/PA whole-spine radiographs; commercially available AI software	196 radiographs from 182 patients; 194 curves included in final performance assessment; reference standard based on four radiologists	End-vertebra selection accuracy 88.6%; mean difference 0.16°; MAD 2.47°; ICC 0.97; processing time below 10 seconds	Multiple expert readers and agreement analysis strengthen the reference standard. The cohort was relatively small, without external or temporal validation.	Users considered the system intuitive, indicating potential workflow feasibility. The publication described commercial availability but did not establish regulatory approval

				Incorrect end-vertebra selection remained clinically relevant despite high ICC	specifically for scoliosis, cost-effectiveness, or downstream effects on care. **Readiness: small clinical validation study**
**Casabó-Vallés et al. [24]**	Fully autonomous detection of spinal curves and Cobb angle measurement on AP/PA radiographs; two-model SPARC pipeline	Retrospective multicentre cohort from four tertiary hospitals; 484 radiographs and 1,054 consensus curves	Curve-detection rate 94.0%; MAE 3.01° ± 2.71°; error range −14.6° to 20.3°	Multicentre data and consensus review by several clinicians are major strengths. However, data were retrospectively curated, temporal validation was not reported, and occasional large individual errors remained despite acceptable mean performance	Demonstrates stronger generalisability than most single-centre systems. Prospective PACS-based deployment, clinician-correction frequency, regulatory status, and economic value were not evaluated. **Readiness: multicentre retrospective validation**
**Chen et al. [25]**	Cobb angle estimation and severity classification	3,899 full-spine radiographs from seven hospitals; two hospitals used	Mean errors below 4° and R^2 >0.90 across all external cohorts; sensitivity	Strongest evidence of cross-centre technical generalisability in	Real-time transformation may facilitate plug-and-play

	across heterogeneous radiographic sources; SpineHRNet+ with real-time pixel-intensity transformation	for development/internal validation and five external cohorts comprising 865 images	90.2% and NPV 93.2% for severity grading; 95% limits of agreement within 7.51°	the chapter. External datasets were genuinely centre-separated and imaging heterogeneity was explicitly addressed. Study remained retrospective; temporal validation, probability calibration, and prospective human–AI interaction were not assessed	deployment across imaging systems. However, prospective workflow benefit, regulatory status, threshold-related decision errors, and cost-effectiveness remain unknown. **Readiness: multicentre external validation**
**Maeda et al. [26]**	Automated measurement of major and minor Cobb angles on standing, supine, bending, and brace radiographs; two-stage ResNet34-based landmark detection	915 images for training, 106 for testing, and 155 for clinical evaluation; single-centre retrospective data	ICC 0.973 for the major curve on standing radiographs; AI measurements were approximately 1.7°–2.2° lower than clinician measurements; lower agreement for the second minor curve on	Evaluation across several clinically relevant positions and comparison with six clinicians are strengths. External validation was absent, and postoperative, congenital, adult-degenerative, and some external-brace images were	Potentially useful for follow-up and flexibility assessment. Performance variability by curve and image type requires clinician oversight. No workflow, regulatory, or economic evaluation.

			bending images	not represented	**Readiness: broad single-centre validation**
**Kang et al. [27]**	Automated measurement of 15 sagittal alignment parameters from lateral full-spine radiographs; RTMpose landmark detection	560 radiographs from five hospitals; 500 used for development/internal validation and 60 from a separate hospital for external validation	ICCs 0.892–0.991; angular MAEs 0.22°–3.32°; SVA MAE 1.16 mm; 91%–100% of keypoints within 4 mm of the reference standard	Independent external-centre testing and evaluation of multiple parameters strengthen the evidence. The external sample was small, restricted to adolescents with AIS, and temporal validation and failure analysis were limited	Rapid automated multiparametric measurement may improve reporting efficiency. Prospective workflow effects, clinician-correction rates, regulatory status, and health-economic outcomes were not evaluated. **Readiness: external technical validation**
**Liu et al. [28]**	Automated measurement of 10 coronal-plane parameters on full-length AP radiographs; multiresolution VB-Net-based commercial system	Retrospective single-centre cohort of 2,092 patients with AIS; images acquired using Siemens and GE systems; no independent external validation	The study reported associations between AI-derived parameters and sex, curve severity, and curve type rather than a complete independent accuracy analysis	Large cohort and multiparametric assessment are strengths. However, using AI-generated measurements for downstream analysis without comprehensive independent	Potentially useful for large datasets and comprehensive deformity description, but measurement validity across centres and postoperative cases remains

			against manual measurements	validation creates uncertainty regarding measurement bias. Postoperative cases were excluded; temporal validation and failure rates were not reported	insufficiently established. Regulatory and economic evidence were not reported. **Readiness: retrospective analytical use of an AI measurement system**
**Du et al. [29]**	Vertebral landmark detection, Cobb angle calculation, and automated Lenke classification; LAD-Net with adjacent-centre correction and corner-feature optimisation	Development and testing dataset size should be extracted from the full text; comparative technical validation without reported prospective or temporal evaluation	Improved landmark localisation, Cobb angle estimation, and Lenke classification compared with benchmark architectures; clinically interpretable error and failure-frequency data require full-text confirmation	Explicit use of adjacent-vertebra structure may reduce landmark outliers. Clinical appraisal is limited by incomplete reporting in the current draft regarding patient selection, patient-level splitting, external testing, and errors near Lenke classification boundaries	Potential relevance to surgical classification, but no prospective treatment-planning study, workflow integration, regulatory information, or economic evaluation. **Readiness: technical internal validation**
**Lang et al. [30]**	Automated coronal and	555 paired examinations from	Complete generation of all	Transparent reporting of	Some successful measurements

	sagittal spinopelvic measurement from paired EOS radiographs; modified U-Net models with inception modules	393 uninstrumented patients; 455 used for training and 100 previously unseen cases for testing	parameters in only 45% of test cases; Cobb-angle MAD 3.3°; 80% of Cobb measurements within ±5°; 85% of SVA measurements within ±5.7 mm	pipeline failures is a major strength. High incomplete-processing rate, difficulty in the thoracic region, and exclusion of instrumentation limit routine applicability. Single-centre data and no temporal validation	were clinically acceptable, but failure in more than half of cases prevents autonomous use. No prospective workflow, regulatory, or cost-effectiveness evaluation. **Readiness: proof of concept with substantial technical failure rate**
**Li et al. [15]**	Three-dimensional spinal segmentation and 3D Cobb angle estimation from preoperative CT; 3D U-Net combined with NURBS-net	116 patients with AIS or degenerative scoliosis; internal validation included 27 patients with AIS; retrospective single-centre study	Correlation r=0.983 with surgeon-measured standing radiographic Cobb angles; MAE 2.4°; Bland–Altman limits −4.0° to 6.2°	Patient-specific 3D analysis and agreement analysis are strengths. CT and reference radiographs were acquired in different positions, and no independent true 3D reference standard was used. The validation cohort	Potential use in preoperative planning, but additional CT solely for AI measurement would raise radiation and cost concerns. No prospective decision-impact, workflow, regulatory, or economic evaluation.

				was small and did not include congenital scoliosis	**Readiness: internal 3D measurement validation**
**van der Graaf et al. [14]**	Automated coronal Cobb angle measurement from routine sagittal lumbar MRI; 3D vertebral and disc segmentation followed by plane fitting	Segmentation model developed using 447 MRI examinations; validation cohort of 50 patients with degenerative scoliosis	ICC 0.83 for the automatically selected maximum angle; ICC 0.92–0.97 when calculated at reader-selected levels; all readers selected identical end levels in only 18% of cases	Demonstrates the reproducibility advantage of algorithmic level selection. Small retrospective cohort, supine MRI acquisition, limited field of view, and lack of direct comparison with standing whole-spine radiographs restrict interchangeability	May reduce additional radiographs when suitable MRI already exists, but cannot yet replace standing radiography for longitudinal monitoring. Regulatory, workflow, and economic implications were not assessed. **Readiness: small internal validation**
**Bassani et al. [31]**	Generation of synthetic sagittal EOS radiographs from coronal images; two sequential pix2pix-based GANs	3,935 paired EOS examinations for development; 100 randomly selected test cases, of which only 69 synthetic images were assessable by both radiologists	Correlations with real images: lumbar lordosis r=0.52, sacral slope r=0.17, pelvic incidence r=0.18, and SVA r=0.74; SVA MAE 1.1±0.8 cm	Large development dataset but substantial test-stage failure: 31% of images were not measurable. Severe curves, postoperative cases, vertebral deformities, and	Potential radiation reduction remains hypothetical because the synthetic projection cannot yet replace an acquired lateral image. No prospective safety,

				nonstandard positioning were excluded. Quantitative accuracy was inadequate for most parameters	regulatory, workflow, or cost-effectiveness evaluation. **Readiness: experimental feasibility only**
**Lee et al. [32]**	Opportunistic scoliosis detection on PA chest radiographs; StyleGAN2-ADA representation learning followed by an MLP classifier	Retrospective data from two centres; 3,587 collected images, with exact Cobb labels available for 16%; 2,163 scoliosis images used for representation learning; artificially balanced test set	AUC 0.856; sensitivity 98.5%; NPV 95.0%; specificity 28.5%; PPV 57.9%	High sensitivity was achieved at the expense of many false-positive alerts. Artificial test prevalence limits interpretation of predictive values. Other chest abnormalities, poor-quality images, and complex postoperative cases were excluded. Calibration and prospective validation were not reported	May support opportunistic case finding, but low specificity could generate substantial downstream radiography and referral costs. No workflow-impact, regulatory, or formal economic evaluation. **Readiness: retrospective screening proof of concept**
**Xie et al. [33]**	Vertebral segmentation, Cobb angle	96 images used for development/validation and 493 for	Mean AI–expert difference 0.40°±0.87°; ICC	Detailed vertebral-level and diagnostic metrics	Promising for opportunistic detection, but

	measurement, and scoliosis screening on chest radiographs; supervised Mask R-CNN module with rule-based inference	testing; 491 images included in final analysis; single-source dataset	0.915; accuracy 98.4%, specificity 98.7%, sensitivity 88.2%, and AUC 0.979 for scoliosis detection	were reported. However, only 17 scoliosis cases were present in the test set, making accuracy strongly influenced by class imbalance. External validation and calibration were absent; severe and instrumented cases were underrepresented	false-negative performance is imprecisely estimated because of the very small positive sample. No prospective workflow, regulatory, or cost analysis. **Readiness: limited retrospective validation**
**Govindarajan et al. [34]**	Simultaneous detection of scoliosis and multiple other abnormalities on chest radiographs; commercial qXR deep-learning system	Prospective multicentre quality-improvement study across 35 centres; 65,604 radiographs processed and 48,940 included in the final performance analysis	AUC approximately 0.98 and NPV 99.9% for radiographs labelled as showing scoliosis	Large prospective real-world deployment is a major strength. However, the reference standard was the routine radiological report rather than dedicated Cobb measurement or standing whole-spine imaging. High NPV may partly reflect low prevalence.	Demonstrates actual workflow implementation of a multipathology chest-radiograph system, but not definitive validation for scoliosis diagnosis. Regulatory status specifically covering scoliosis and health-economic consequences

				Scoliosis severity and treatment-threshold errors were not evaluated	were not established in the study. **Readiness: prospective implementation evidence for opportunistic detection**
**Kabir et al. [38]**	Automated measurement of magnetically controlled growing-rod length on postoperative PA radiographs; deep-learning detection and measurement system	387 radiographs for model development; final test set of 60 radiographs containing 118 rods	Mean absolute difference 0.98±0.88 mm; ICC 0.90; average processing time 6.1 seconds per rod	Clinically relevant postoperative measurement and comparison with manual assessment are strengths. External-centre validation, device-manufacturer subgroup analysis, temporal testing, and failure handling were not comprehensively evaluated	May improve follow-up efficiency in early-onset scoliosis, but effects on distraction decisions, revision timing, workflow, costs, and regulatory status remain unknown. **Readiness: internal postoperative measurement validation**

Abbreviations: AI, artificial intelligence; AIS, adolescent idiopathic scoliosis; AP, anteroposterior; AUC, area under the receiver operating characteristic curve; CNN, convolutional neural network; CT, computed tomography; EOS, biplanar low-dose radiographic imaging system; ICC, intraclass correlation coefficient; MAE, mean absolute error; MAD, mean absolute difference/deviation; MLP, multilayer perceptron; MRI, magnetic resonance imaging; NPV, negative predictive value; NR, not reported; PA, posteroanterior; PPV, positive predictive value; SVA, sagittal vertical axis.

Methodological note: Calibration was considered primarily relevant to probabilistic diagnostic classification models. For continuous measurement systems, clinical appraisal focused instead on systematic bias, mean absolute error, Bland–Altman limits of agreement, failure rates, and errors capable of crossing clinically relevant diagnostic or treatment thresholds. "Regulatory status not reported" means that the publication did not provide sufficient information regarding approval for the specific scoliosis-related indication; it should not be interpreted as evidence that no approval exists.
