# Supplementary material for: From Algorithms to Clinics: Recent Progress in AI for Scoliosis Diagnosis and Management
Source: J Clin Med. 2026 Aug 18;15(16):6361. doi: 10.3390/jcm15166361 (PMC13513889; doi:10.3390/jcm15166361)
Supplement: Supplementary file 1 [file jcm-15-06361-s001.zip › Supplementary Table S2.pdf]

Supplementary Table S2. Methodological characteristics, validation strategies, performance, and clinical readiness of AI systems for scoliosis screening, deformity classification, automated radiographic measurement, and outcome prediction

Study	Clinical task and input data	Study design and population	AI method	Reference standard or predicted outcome	Validation and calibration	Main results	Principal limitations and current clinical status
**Mohamed et al., 2025**	Radiation-free detection of AIS using three-dimensional markerless surface-topography scans	558 adolescents contributing 991 scans; participants with radiographically confirmed AIS and screen-negative individuals with typically developing spines	Convolutional neural network for binary scoliosis classification	Radiographic confirmation of AIS; control participants were screen-negative rather than systematically confirmed by spinal radiography	Internal held-out test set based on a random scan-level split; no independent external or temporal validation; calibration not reported	Accuracy 95%, sensitivity 97%, specificity 90%, and false-negative rate 2.9%	Binary classification only, without Cobb-angle estimation or severity classification. Repeated scans from individual participants and the scan-level split may have introduced patient-level leakage. Generalisability to unselected screening populations

							and non-standardised acquisition conditions remains uncertain.
**Duan et al., 2025**	Detection and severity stratification of scoliosis from standardised bare-back photographs	458 participants from three hospitals: 350 with scoliosis and 108 controls; pooled random split into training, validation, and test sets	Dual AttentionUNet for back-contour segmentation combined with a computer-vision-derived asymmetry index	Standing radiographic Cobb-angle measurements	Internal validation using a pooled random split; no centre-held-out, temporal, or prospective population-level validation; calibration not reported	AUC 0.927 for scoliosis detection. For severe scoliosis, defined as Cobb angle ≥45°, accuracy was 96.9%, precision 95.6%, sensitivity 92.3%, and AUC 0.954	Images were acquired under controlled conditions using a dedicated camera. Poor-quality photographs were excluded, and performance was reduced in patients with higher BMI or less pronounced deformity. The model classified severity but did not predict

							treatment requirements.
**Martrenchar et al., 2026**	Smartphone-based scoliosis screening using video-derived three-dimensional surface topography	Prospective multicentre diagnostic study of 236 participants, including 154 patients with idiopathic scoliosis and 82 controls, recruited in France and Canada	Smartphone application generating a three-dimensional representation of the back and estimating the major coronal curve	Radiographic Cobb angle, with scoliosis defined using a threshold greater than 10°	Prospective two-centre validation; measurement reproducibility, probability calibration, and clinical implementation impact were not assessed	Sensitivity 100% and specificity 89% for detecting curves greater than 10°	Potential selection bias and a relatively enriched case–control population may overestimate screening performance. The study did not determine whether use of the application reduced radiographic examinations, false-positive referrals, costs, or diagnostic delays. Promising for preliminary

							triage but not yet validated as a replacement for clinical or radiographic assessment.
**Chen et al., 2026**	Prediction of paediatric scoliosis using foot-pressure distribution, balance, and body-angle parameters	School-based dataset of 5,970 children; 3,723 participants remained after exclusion of incomplete or invalid records	Logistic regression, support vector machine, Random Forest, and LightGBM; SHAP used for feature interpretation	Study-defined paediatric scoliosis status used as the classification target	Internal model development and comparison; no independent external or temporal validation; calibration not reported	LightGBM achieved an AUC of 0.971 and accuracy of 92%. Toe-out angle and centre-of-pressure sway area were among the most influential predictors	Considerable exclusion of records may have introduced selection bias. Performance was not evaluated in an independent geographical population or prospective screening workflow. The consequences of false-positive classification, referral thresholds, and the

							incremental value over conventional screening were not assessed.
**Berlin et al., 2024**	Fully automated measurement of coronal deformity parameters on anteroposterior or whole-spine radiographs	Retrospective single-centre validation using 100 preoperative EOS radiographs from patients with AIS	Automated landmark and anatomical-structure detection with calculation of T1 tilt, clavicle angle, coronal balance, lumbar modifier, and regional Cobb angles	Independent measurements by two experienced physicians	Agreement study in a single-centre validation cohort; no external, temporal, postoperative, or cross-device validation; probability calibration not applicable	ICCs between AI and clinicians ranged from 0.78 to 0.98. The lowest agreement was observed for the proximal thoracic Cobb angle. Processing required approximately 15 seconds per image compared with 3–7 minutes for manual assessment	Restricted to preoperative AIS radiographs acquired using one EOS system and to coronal-plane measurements. Performance on conventional radiographs, postoperative images containing implants, sagittal parameters, atypical deformities, and poor-

							quality images was not established.
**Lafage et al., 2023**	Data-driven classification of complex adult spinal deformity using coronal and sagittal radiographic parameters	Retrospective analysis of 286 surgically treated patients with complex adult spinal deformity	Unsupervised cluster analysis	No diagnostic reference standard; clusters were derived from radiographic morphology and examined for associations with perioperative characteristics and outcomes	Internal derivation analysis without independent validation; calibration not applicable	Four deformity phenotypes were identified: hyper-thoracic kyphosis, moderate sagittal deformity, severe sagittal deformity, and predominantly coronal deformity	The clusters were derived from a selected surgical population and were not designed to predict an individual patient's outcome or determine treatment. Applicability to non-operative patients, other centres, and different populations remained uncertain.
**Lafage et al., 2025**	Evaluation of previously defined adult	Retrospective analysis of a prospectively	Assignment of patients to previously	Two-year patient-reported	Application of predefined clusters in a	Patients in the coronal cluster	Restricted to surgically treated adult

	spinal deformity clusters as predictors of postoperative clinical trajectories	maintained multicentre database; 1,062 surgically treated patients with complete two-year follow-up from 1,476 eligible cases	established radiographic cluster centroids	outcomes, achievement of MCID, disability, major complications, and complications requiring reoperation	separate, larger multicentre cohort; no calibrated individual-level risk model or prospective decision-impact evaluation	achieved MCID in the SRS-22 appearance domain more frequently than those with moderate sagittal deformity, 77.9% versus 58.0%. Severe sagittal deformity was associated with the greatest residual disability and highest complication burden	deformity and affected by incomplete two-year follow-up. Observed associations may reflect baseline differences and treatment selection. The clusters have not been validated as a tool for selecting procedures or estimating individual treatment benefit.
**Vu-Han et al., 2025**	Identification of scoliosis-related risk patterns in patients with treated 5q-	84 patients with genetically confirmed 5q-SMA, contributing	Expert-guided feature engineering followed by a Random	Scoliosis defined primarily as Cobb angle >10°, although	Repeated stratified patient-grouped cross-validation,	Mean accuracy 0.82, precision 0.85, and ROC AUC	Small single-centre cohort and heterogeneous outcome ascertainmen

	spinal muscular atrophy using longitudinal clinical data	1,304 clinical visits and more than 360 variables; 19 patients with uncertain scoliosis status were excluded from modelling	Forest classifier	labels were derived from heterogeneous radiographic and clinical sources	preventing visits from the same patient from appearing in both training and test data; no external or temporal validation; calibration not reported	0.87	t. Dedicated spinal radiographs were available for only 41 patients. The model primarily identified scoliosis status or associated risk patterns rather than prospectively predicting future onset within a defined time horizon.
**De la Garza Ramos et al., 2022**	Prediction of perioperative red blood cell transfusion in adult spinal deformity surgery	Retrospective analysis of 1,173 patients recorded in the 2017–2019 ACS-NSQIP databases	Artificial neural network using 18 patient-related and operative variables; optimal architecture	Red blood cell transfusion occurring intraoperatively or within 72 hours after surgery	Internal random 70:30 train–test split; no independent external or temporal validation; calibration not reported	Sensitivity 0.80, positive predictive value 0.76, F1 score 0.78, accuracy 0.77, and AUROC 0.84	No direct comparison with a conventional statistical model was performed. Some predictors, particularly

			contained hidden layers of 128 and 64 neurons				actual operative duration, may not be available preoperatively. Specificity and negative predictive value were not reported, and the effect on blood-product allocation, complications, or costs was not evaluated.
**Shi et al., 2025**	Prediction of patient-reported and radiographic outcomes after posterior spinal fusion for AIS	Multisite cohort of 455 patients from two paediatric hospitals; 428 had baseline and postoperative data, including 378 at 6 months,	Gaussian naïve Bayes, logistic regression, Random Forest, support vector machine, XGBoost, and a multilayer perceptron	Individual SRS-22R responses, achievement of MCID in SRS-22R domains, and a composite outcome combining patient-reported	Patient-level 80:20 train–test split with five-fold cross-validation in the training set; calibration was assessed and adjusted; no	Random Forest and support vector machines achieved average AUROCs of approximately 0.81–0.86 for individual SRS-22R	Retrospective analysis with decreasing sample size at longer follow-up and numerous predictors and outcomes, increasing the risk of

		351 at 1 year, and 260 at 2 years	using 171 preoperative variables; SHAP used for interpretation	improvement with >60% major Cobb-angle correction	centre-held-out or temporal validation	question prediction. The deep-learning model achieved overall AUROCs of approximately 0.86, 0.84, and 0.83 at 6 months, 1 year, and 2 years	overfitting. Data from both centres were pooled before splitting. Prospective effects on counselling, treatment selection, rehabilitation, or patient outcomes were not assessed.

Abbreviations: AIS, adolescent idiopathic scoliosis; ASD, adult spinal deformity; AUC/AUROC, area under the receiver operating characteristic curve; BMI, body mass index; ICC, intraclass correlation coefficient; MCID, minimal clinically important difference; SRS-22R, revised 22-item Scoliosis Research Society questionnaire; SMA, spinal muscular atrophy; SHAP, SHapley Additive exPlanations; ACS-NSQIP, American College of Surgeons National Surgical Quality Improvement Program.

Note: Studies already described in detail in the preceding section and included in Supplementary Table S1, including Zhang et al., Lee et al., and Kang et al., were not repeated in this table. This approach avoids duplication while allowing the studies to be briefly cross-referenced in the narrative where relevant.
