# Supplementary material for: From Algorithms to Clinics: Recent Progress in AI for Scoliosis Diagnosis and Management
Source: J Clin Med. 2026 Aug 18;15(16):6361. doi: 10.3390/jcm15166361 (PMC13513889; doi:10.3390/jcm15166361)
Supplement: Supplementary file 1 [file jcm-15-06361-s001.zip › Supplementary Table S3.pdf]

Supplementary Table S3. Methodological characteristics, validation strategies, performance, and clinical readiness of predictive models in scoliosis and related spinal disorders

Study	Clinical task and input data	Study design and population	Model and predicted outcome	Validation and calibration	Main results	Principal limitations and clinical readiness
**Lv et al., 2023 [51]**	Non-invasive identification of existing AIS using anthropometric and postural examination variables	Retrospective two-centre study of 3,313 children and adolescents; 1,732 participants in the development cohort and 1,581 in a geographically separate validation cohort	Random Forest, support vector machine, artificial neural network, decision tree, and generalized linear model; outcome was the presence of AIS	Internal development and validation plus validation in a cohort from a separate hospital; decision-curve analysis performed; comprehensive probability calibration not reported	The ANN achieved AUCs of 0.899 in the training dataset and 0.897 in the internal validation dataset and remained the highest-performing model in the separate-hospital cohort	The model detected AIS already present at assessment rather than predicting future onset or progression. Predictors largely represented visible manifestations of trunk deformity. Radiographic verification was not uniform across all screened participants, creating a risk of partial verification

						bias. Relevant to screening, not prognosis.
**Fang et al., 2025 [8]**	Prediction of continued AIS progression using longitudinal radiographic features automatically extracted from serial full-spine radiographs	Multicentre retrospective study of 233 patients from three centres; 183 patients from one centre used for model development and internal testing and 50 patients from two additional centres used for external validation	Logistic regression, SVM, decision tree, Random Forest, XGBoost, and K-nearest neighbours; progression defined as an increase of >5° in the major Cobb angle between the first and third examinations	Internal patient-level testing and geographically external validation; no temporal or prospective validation; calibration and decision-curve analysis not reported	XGBoost achieved an external-validation AUC of 0.896, accuracy of 0.88, sensitivity of 1.00, and specificity of 0.857. SHAP identified early change in the major Cobb angle as the most influential predictor	The model required at least two previous radiographic examinations and therefore predicted continued progression after an early trajectory had already been observed. The external cohort included only 50 patients and eight progression events. SHAP demonstrates model attribution rather than causality. Preliminary dynamic risk model, not yet

						ready to determine follow-up or bracing decisions.
**Johnson et al., 2023 [52]**	Preoperative prediction of radiographic proximal junctional kyphosis within two years after adult spinal deformity surgery using clinical, radiographic, and MRI data	Retrospective single-centre cohort of 191 patients, of whom 89 developed PJK; preoperative thoracic MRI was available for only 59 patients	SVM using structured demographic, operative, and radiographic variables; CNN-based models combining structured data with raw scoliosis radiographs or T1-weighted thoracic MRI	Internal nested five-fold cross-validation; no external or temporal validation; calibration and clinical decision analysis not reported	Structured-data model: sensitivity 57.2%, specificity 56.3%; radiograph-enhanced model: sensitivity 68.2%, specificity 58.3%; MRI-enhanced model: sensitivity 73.1%, specificity 79.5%	MRI performance was estimated in a very small subgroup. Grad-CAM highlighted posterior thoracic soft tissues but did not establish a causal role of muscle degeneration. The study did not test whether predictions altered construct planning or preventive strategies. Requires larger prospective multicentre

						validation.
**Ayvaz et al., 2025 [53]**	Prediction of Cobb-angle change and pain reduction after Schroth-based exercise therapy; separate active-learning experiment for curve-direction classification	Retrospective real-world cohort of 128 individuals treated for 3–36 months	Random Forest regression using age, baseline Cobb angle, and treatment duration; uncertainty-based active learning used in a separate classification task	Model-development and validation procedures were incompletely described; no external or temporal validation; calibration not reported	Pain-reduction model achieved MAE 0.84 and RMSE 1.06. Active-learning classification accuracy increased from 65% to 85% over five simulated labelling iterations	The cohort included both adolescents and adults, including older patients, and had a median baseline Cobb angle below the conventional scoliosis threshold. The active-learning result concerned curve-direction classification, not treatment-response prediction. Uncontrolled design, adherence-based exclusions, heterogeneous treatment duration, and reporting inconsistencies

						prevent causal or health-economic conclusions. Exploratory only.
**Park et al., 2025 [54] — contextual spine-surgery evidence**	Prediction of postoperative hospital length of stay using structured electronic health-record variables and operative notes from spine surgery cases	Retrospective EHR-based spine-surgery dataset; not specific to scoliosis or spinal deformity	Eight approaches, including conventional ML, a standalone LLM, retrieval-augmented generation, retrieval-augmented prediction based on similar previous cases, and combined models	Internal evaluation reported; independent geographical or temporal validation and calibration of continuous predictions were not established in the published abstract	Retrieval-augmented prediction alone achieved R^2 0.39 and MAE 4.47 days. A neural network combined with retrieval-augmented prediction achieved the best performance, with R^2 0.52 and MAE 4.16 days	Moderate residual prediction error limits individual discharge forecasting. The population was broader than scoliosis, and effects on bed allocation, discharge planning, costs, or patient outcomes were not prospectively tested. Provides contextual evidence for operational forecasting rather than scoliosis-specific

						prognosis.
**Hosozawa et al., 2026 [55] — contextual spine-surgery evidence**	Prediction of clinically important postoperative improvement in JOABPEQ domains and pain outcomes following lumbar spine surgery	Multicentre study involving 1,149 patients; 981 patients from two centres used for model training and internal validation and 168 patients from a third centre used for external validation	TabNet, deep neural network, and elastic-net penalised logistic regression	Stratified five-fold cross-validation during development and independent centre-level external validation; ROC and calibration curves were evaluated	In external validation, TabNet achieved mean AUC 0.79 and accuracy 0.74; DNN achieved AUC 0.77 and accuracy 0.73; elastic-net logistic regression achieved AUC 0.78 and accuracy 0.74	Deep learning did not clearly outperform penalised logistic regression. The study concerned lumbar degenerative surgery rather than scoliosis. No prospective clinical-impact evaluation demonstrated improvement in patient selection or counselling. Methodologically relevant contextual evidence because of independent external validation and calibration assessment.

**André et al., 2026 [56] — contextual spine-surgery evidence**	Prospective prediction of a composite clinically important outcome after lumbar decompression surgery using preoperative and perioperative variables	Prospective multicentre study; 119 patients were enrolled, 103 provided postoperative follow-up, and the principal prediction analysis included 61 patients with more than 90 days of follow-up	Previously developed artificial neural network predicting MCID versus no MCID; MCID combined ODI, motor function, and medication-use criteria	Prospective evaluation in real patients, but with a small complete-case cohort; no additional independent external or temporal validation; calibration and decision-curve analysis not reported	AUC 0.816, accuracy 70%, sensitivity 81%, specificity 59%, PPV 68%, and NPV 74%	The abstract describes 81.6% as “accuracy,” whereas the detailed results identify 0.816 as the AUC and report accuracy of 70%. The model had previously been trained partly on 10,000 synthetic records and tested on 2,000 synthetic records. Moderate specificity and the small real-world test cohort limit transportability. Not scoliosis-specific.
**Vila et al., 2026 [57]**	Prediction of achieving a patient-acceptable	Retrospective analysis of a prospective multicentre	Separate Random Forest models for three validated	Phenotype-specific internal model evaluation	AUC ranged from 0.73 to 0.94 depending on phenotype.	Prediction performance varied substantially

	symptom state in the SRS-22 self-image domain two years after adult spinal deformity surgery	registry including 710 surgically treated patients with at least two years of follow-up	adult spinal deformity phenotypes: Young Coronal, Old Moderate, and Old Severe	within a multicentre registry; independent external and temporal validation and calibration metrics were not reported in the abstract	Functional disability, pain, and physical and social functioning were more influential than radiographic parameters or complications	between phenotypes, and cluster-specific sample and event sizes may affect model stability. The retrospective analysis did not demonstrate that predicted self-image outcomes improve shared decision-making. Clinically relevant because it prioritises patient-centred outcomes, but prospective impact evaluation remains necessary.

Abbreviations: AIS, adolescent idiopathic scoliosis; ANN, artificial neural network; AUC, area under the receiver operating characteristic curve; CNN, convolutional neural network; DNN, deep neural network; EHR, electronic health record;

JOABPEQ, Japanese Orthopaedic Association Back Pain Evaluation Questionnaire; LLM, large language model; MAE, mean absolute error; MCID, minimal clinically important difference; MRI, magnetic resonance imaging; NPV, negative predictive value; ODI, Oswestry Disability Index; PASS, patient-acceptable symptom state; PJK, proximal junctional kyphosis; PPV, positive predictive value; RMSE, root mean square error; SHAP, SHapley Additive exPlanations; SRS-22, 22-item Scoliosis Research Society questionnaire; SVM, support vector machine.

Notes: Lonstein and Carlson [64] were included as a historical comparator rather than an AI model. Lv et al. [65] were retained because the study is discussed in the narrative, but the model classified existing AIS and should not be interpreted as predicting future progression. Park et al. [70], Hosozawa et al. [71], and André et al. [72] investigated broader spine-surgery populations and are included as contextual evidence for operational and patient-reported outcome prediction. Vu-Han et al. [61] were not repeated because the study is presented in the preceding section and supplementary table.
