# Supplementary material for: From Algorithms to Clinics: Recent Progress in AI for Scoliosis Diagnosis and Management
Source: J Clin Med. 2026 Aug 18;15(16):6361. doi: 10.3390/jcm15166361 (PMC13513889; doi:10.3390/jcm15166361)
Supplement: Supplementary file 1 [file jcm-15-06361-s001.zip › Supplementary Table S4.pdf]

Supplementary Table S4. Methodological characteristics, validation strategies, performance, and clinical readiness of artificial intelligence systems for scoliosis surgical planning and postoperative care

Study	Clinical task and input data	Study design and population	AI method	Reference standard or predicted outcome	Validation and calibration	Main results	Principal limitations and current clinical readiness
**Dong et al., 2025**	Automated preoperative Cobb-angle measurement and Lenke classification using standing AP, lateral, left-bending, and right-bending full-spine radiographs	Cross-sectional study; 506 patients with AIS contributing 1,812 radiographs for retrospective model development; an additional 107 patients were prospectively included for testing	Multiple-view U-Net semantic segmentation with automated vertebral alignment, Cobb-angle extraction, and rule-based Lenke classification	Manual radiographic measurements and Lenke classifications performed by clinicians	Prospective testing in an additional cohort from the same clinical setting; no independent geographical or temporal validation; probability calibration not applicable and no decision-impact analysis	Agreement coefficients were 0.989 for Lenke curve type, 0.932 for the lumbar modifier, and 0.987 for the sagittal modifier. After manual review of discordant cases, reported accuracy was 92%, 91%, and 89%, respectively. Automated processing required	Lenke type 4 and positive sagittal modifiers were underrepresented, preventing reliable assessment of all classification categories. The study evaluated classification consistency rather than the appropriateness of fusion-level selection or

						approximately 30 seconds compared with approximately 3 minutes for manual assessment.	postoperative outcomes. The model may standardise preoperative classification but is not a complete surgical-planning system.
**Xu et al., 2026**	Fully automated Lenke classification from AP and lateral full-spine radiographs	Retrospective study of 650 individuals, including 467 patients with AIS and 183 controls; AIS curves ranged from 25° to <90°	Multistage system combining Swin-Unet vertebral segmentation, DeepLabv3+ lumbar-pedicle localisation, automated Cobb-angle calculation, and a feature-fusion classification module	Expert Lenke classification and manual Cobb-angle measurements	Independent internal test set; no centre-held-out external or temporal validation; model calibration and clinical-impact assessment not reported	Overall Lenke-classification accuracy was 95.6%, with a macro-averaged F1 score of 0.862. Cobb-angle ICCs ranged from 0.969 to 0.976. F1 scores were 0.912 for the lumbar modifier and 0.928 for the sagittal	The study population was predominantly Han Chinese and came from a limited institutional setting. The system classified deformity but did not recommend fusion levels, simulate correction, or evaluate

						modifier.	effects on surgical decisions and outcomes. High internal accuracy does not establish transportability or clinical utility.
**Phellan Aro et al., 2024**	Real-time simulation of postoperative three-dimensional spinal shape for alternative correction targets	Sixty-four patients with AIS were used to generate 6,400 finite-element biomechanical simulations	Dimensionality reduction using an autoencoder and principal component analysis followed by machine-learning regression; PCA combined with linear regression performed best	Outputs generated by patient-specific finite-element simulations	Internal simulation-based evaluation; no comparison with actual postoperative anatomy, independent centres, or prospective clinical decisions; calibration not applicable	Mean vertebral-position error was 3.75 mm, with orientation errors below 2.74° in all three principal axes. Predictions were produced in approximately 3 ms, compared with seconds or minutes	The model learned to reproduce simulated finite-element outputs rather than observed postoperative results. Any errors or assumptions in the biomechanical model may therefore be inherited by the ML

						for conventional finite-element simulations.	surrogate. Clinical usefulness requires validation against actual postoperative alignment and prospective assessment during surgical planning.
**He et al., 2026 — ScoliosisPLAN**	Integrated selection of upper and lower instrumented vertebrae, estimation of total fusion length, and generation of predicted postoperative coronal radiographs	Multicentre study of 1,425 surgically treated patients with AIS and ≥2-year follow-up: 1,043 training cases, 100 validation cases, 200 prospectively recruited internal test	ScolioPlanNet, a YOLOv8-derived segmentation and fusion-planning network incorporating standing and bending radiographs; ScolioPredNet, a conditional latent diffusion	Fusion levels implemented by surgeons; actual postoperative radiographs and measured postoperative major and secondary Cobb angles and coronal balance	Retrospective development with prospective internal and external validation at three centres; Bland–Altman and regression agreement analyses performed for postoperative	Total fusion-length MAE was 0.34 vertebral levels internally and 0.63 and 0.52 in the two external cohorts. An experienced surgeon achieved an MAE of 0.30 and a less-experienced	The system reproduced fusion strategies selected in previous clinical practice rather than identifying an independently proven optimal construct. Forty-two patients with

		cases, and two prospective external test cohorts of 38 and 44 patients	model generating predicted postoperative radiographs		predictions; formal probability calibration not applicable	surgeon 0.67. In the internal prospective cohort, postoperative prediction MAEs were 2.94° for the major Cobb angle, 2.60° for the secondary Cobb angle, and 2.92 mm for coronal balance; performance remained broadly stable in external cohorts.	postoperative coronal complications were excluded from the primary fusion-planning analysis; the model reproduced the original surgical plan in 28 of these cases. Agreement with historical decisions therefore does not demonstrate motion-segment preservation, prevention of complications, or improved outcomes. Prospective

							comparative impact studies are required before clinical deployment.
**Jia et al., 2026**	Selection of upper and lower instrumented vertebrae from structured clinical and radiographic information	Preliminary retrospective study of 203 consecutive patients with AIS meeting surgical indications	DeepSeek-R1 reasoning-oriented large language model provided with standardised textual prompts containing clinical information, radiographic measurements, and Lenke classification	Independent ratings of surgical reasonableness by three experienced spine surgeons using a five-point Likert scale	Internal expert-rating study; no independent patient cohort, temporal validation, calibration, or postoperative outcome assessment	Proposed fusion levels were rated as surgically reasonable in 70.9% of cases. Performance was relatively high for Lenke 1A, 1B, 5C, and 6C, but substantially lower for 1C, 2A, and 2C, with acceptable recommendations in 19.0%, 42.9%, and 25.0% of cases,	The model did not directly interpret raw radiographs and was provided with pre-extracted measurements and Lenke classification. The reference standard was subjective expert assessment of reasonableness rather than actual surgical plans or long-term

						respectively. Inter-rater ICC was 0.840.	outcomes. Marked subtype-dependent performance precludes autonomous use, particularly in complex curves.
**Aktan et al., 2025**	Direct multimodal interpretation of standing and side-bending radiographs for Lenke classification and fusion-level recommendation	Retrospective single-centre study of 125 surgically treated patients with AIS, providing four radiographs per patient	Four general-purpose multimodal LLMs: ChatGPT, Claude, Gemini, and DeepSeek; identical prompts were repeated to assess test–retest reliability	Consensus assessment by experienced spine surgeons	Internal comparison with expert consensus and repeated-model testing; no external or temporal validation and no assessment of postoperative outcomes	Agreement with expert consensus ranged from 1.6% to 10.2% for Lenke classification and from 0.8% to 12.0% for fusion-level selection. Cohen's κ values were close to zero, and test–retest reproducibility was	The models demonstrated both poor accuracy and pronounced internal inconsistency. Their rapid response time did not compensate for unreliable radiographic interpretation. General-purpose multimodal LLMs should not currently be used for

						approximately chance level.	AIS classification, fusion planning, or patient self-assessment without task-specific development and validation.
**Lan et al., 2026**	Automated calculation of multiple versions of the Global Alignment and Proportion score and prediction of mechanical complications after adult degenerative scoliosis surgery	Vertebral-detection model developed using 3,485 spinal radiographs with and without instrumentation; GAP-score variants assessed in a retrospective surgical cohort of patients with adult degenerative	Deep-learning vertebral-detection and sagittal-parameter extraction system integrated with editable original and modified GAP-score calculations	Manually assessed sagittal parameters and postoperative mechanical complications	Internal model development and retrospective clinical validation; no independent geographical or temporal validation reported; calibration and decision-curve analysis not reported	Mean average precision was 0.780 for radiographs containing screws and 0.732 for images without screws. The ethnicity-, age-, and sex-adjusted EAGA C-GAP version achieved the highest discrimination for	Although automated measurement may improve efficiency and reproducibility, an AUC of 0.627 represents only modest prognostic discrimination. The system's measurement performance should not be

		scoliosis				mechanical complications, with an AUC of 0.627.	conflated with accurate complication prediction. Clinical thresholds, false-positive consequences, calibration, and prospective effects on alignment planning were not assessed.
**Sundrani et al., 2025**	Prediction of mechanical complications following adult spinal deformity surgery using alignment, bone-quality, and paraspinal soft-tissue variables	Retrospective cohort of 231 patients undergoing fusion of at least five levels with ≥2-year follow-up; 147 patients experienced at least one mechanical complication	Seven machine-learning models using different combinations of demographic variables, postoperative coronal and sagittal alignment, bone-density measuremen	Occurrence of postoperative mechanical complications	Internal model evaluation; no independent external or temporal validation; calibration and decision-curve analysis not reported	The model combining alignment, bone quality, and soft tissue achieved a PPV of 0.90, sensitivity of 0.67, and specificity of 0.84, outperforming models based on	The model incorporated postoperative alignment, limiting its use as a purely preoperative planning tool. Mechanical complications occurred in 64% of the cohort, which substantially

			ts, and paraspinal muscle quantity and fatty infiltration			alignment alone.	influenced the high PPV and may limit transportability to lower-risk populations. The study did not demonstrate that model-guided interventions reduce complications.

Abbreviations: AIS, adolescent idiopathic scoliosis; AP, anteroposterior; AUC, area under the receiver operating characteristic curve; CNN, convolutional neural network; GAP, Global Alignment and Proportion; ICC, intraclass correlation coefficient; LIV, lower instrumented vertebra; LLM, large language model; MAE, mean absolute error; mAP, mean average precision; PCA, principal component analysis; PPV, positive predictive value; RMSE, root mean square error; UIV, upper instrumented vertebra.

Note: Automated measurement studies already discussed in the preceding imaging section were not repeated. The present table includes only systems directly addressing preoperative deformity classification, fusion planning, postoperative alignment simulation, or mechanical-complication assessment. High agreement with historical expert decisions or retrospective annotations should not be interpreted as evidence that a system identifies the optimal surgical strategy or improves patient outcomes.
